# Adenosine receptors on the immuno-oncology expressway: TIME, perspectives, and translation

**DOI:** 10.3389/fimmu.2025.1676702

**Published:** 2026-01-08

**Authors:** Biswanath Majumder, Santanu Datta

**Affiliations:** Bugworks Research India Pvt. Ltd., Centre for Cellular & Molecular Platforms, National Centre for Biological Sciences, Bangalore, India

**Keywords:** adenosine receptors, immune checkpoint resistance, tumor immune microenvironment, emerging vulnerabilities, terminal exhaustion, clinical trials, smart delivery, cancer vaccines

## Abstract

A decade since immune checkpoint inhibitors made a stride in the clinical landscape of oncology, there has been a substantial focus on understanding the response heterogeneity following these therapies. Insights gained from clinical data identified the primary and secondary resistance mechanisms that escape the upfront therapy pressure. Beyond PD-1 and CTLA-4, new checkpoints averting this pressure are under clinical development. Adenosinergic pathways are actively engaged in oncogenic signaling. The main protagonists, CD73, A2AR, and A2BR, span diverse immune subsets of lymphoid and myeloid lineages and have emerged as alternative checkpoints. This review discusses the latest update on immune regulation dynamics of adenosine receptor signaling and their complex interplay with hypoxia in a heterogeneous tumor immune microenvironment (TIME). In this spectrum, we also review the plasticity of A2AR and A2BR in designing new drug candidates, tracing their complex metabolic roots in inducing immune dysfunction. Beyond the existing modalities, the ENT1 and MTAP-loss-MTA axis shows scope for alternative perturbations. The CD39-CD73-A2AR axis plays a central role in the terminal exhaustion of T cells. We highlight the interventions that disrupt the mechanistic context of A2AR and its cooperativity with other suppressors to restore anti-tumor immune functions following inhibition of their multilayered signaling. We capture the ongoing clinical trials and predictive biomarker landscape, along with novel delivery methods, to illustrate the evolving trends in this field. From these perspectives, we discuss how the adenosine axis can widen this new therapeutic avenue and boost the efficacy of CAR-T therapies. Therapeutic cancer vaccines are a new modality in this premise. Finally, an integrated overview of this pathway, along with TIME dynamics, illustrates the barriers and opportunities of combining adenosine signaling inhibitors in clinical trials.

## 1 Introduction

Adenosine receptors belong to the class A family of G-protein-coupled receptors (GPCRs) superfamily with ubiquitous distribution (1). These receptors feature seven transmembrane domains (TMDs), represented by helices TM1 through TM7. They also contain three extracellular loops (ECLs 1, 2, and 3) and three intracellular loops (ICLs 1, 2, and 3) (2). Upon engagement with adenosine, an intermediate metabolite of ATP, they transduce signals through cAMP and PKA. A1, A2A, A2B, and A3 are four molecularly characterized adenosine receptor subtypes. They display differential affinity to adenosine. A2AR has high affinity (30nM), A1R and A3R show intermediate affinity (100 nM), and A2BR is known for low affinity (1000 nM) (3, 4). Depending on the physiological regulation and context, they act through divergent heterotrimeric G-protein coupling. While A2AR and A2BR activate Gαs to transduce signal mainly through cAMP-PKA, A1R and A3R engage Gαi/o in inhibiting second messenger. In some cases, Gβγ subunits transduce signals through mitogen-activated protein kinase (MAPK) and phospholipase Cγ (PLCγ) (4). The pathophysiology of adenosine receptor signaling encompasses a diverse disease landscape that affects all major organ systems and biological processes (5–9). Drugs targeting these action mechanisms are either approved or under clinical development (10). Istradefylline is the first A2A adenosine receptor (A2AR) antagonist clinically approved for Parkinson’s disease (11). During the acute phase of inflammation, adenosine signaling prevents unrestricted immune activation after the clearance of infection, allowing the injured tissue to heal and restore homeostasis. During chronic inflammation, this balance is disrupted, and, despite high adenosine signaling, inflammation persists, leading to the initiation of DNA damage and the development of oncogenic mutations (12). Adenosine receptors, primarily A2AR and A2BR, at the tumor-immune interface, play a crucial role in immune evasion. They misguide the host-defence strategy originally evolved to protect healthy tissues from recurrent damage following inflammation. The enzymes and receptors of the adenosinergic axis polarize the same machinery to safeguard tumors, thus presenting enormous therapeutic challenges. Recognizing these hurdles, there has been an intense focus in recent years on understanding the roles of adenosine (Ado) and its receptors in cancers (11, 13, 14). Early research on Ado-mediated T-cell suppression revealed its *in vitro* effects on the growth of lymphoma (15). Further recognition of A2A adenosine receptor (A2AR) deficiency in promoting autoimmune diseases, and the association of A2B adenosine receptor (A2BR) overexpression with poor prognosis and survival in cancer, opens the door for pioneering new drugs targeting this pathway (16, 17). This appears more pertinent at a time when improved understanding of primary and secondary resistance mechanisms to anti-programmed death 1 (anti-PD-1) therapies reiterates the need to untangle alternative checkpoints and target immunosuppressive metabolic barriers (18, 19). Results from initial studies support the notion that A2AR is a hijacked immune checkpoint, leading to immune suppression (20–22). Harnessing this momentum, A2AR and A2BR inhibiting agents are in the race to determine novel immunotherapy combinations (23).

This review elaborates on the emerging landscape of adenosine signaling in the context of immune checkpoint development. We delve into the intricacies of its multilayered and divergent spread within the tumor immune microenvironment (TIME). Targeting upstream vulnerabilities, such as MTAP loss and diminishing eAdo levels, through ADA and ENT1 is a key part of improving the druggability. We present emerging paradigms, such as terminal exhaustion and its contribution to immune evasion, from an adenosinergic perspective. We further shed light on the conceptual progress and current knowledge gaps regarding the clinical translation of these approaches. Besides elaborating on the scopes of A2AR and A2BR antagonism in improving adoptive immunotherapies, such as CAR-T, we illustrate the prospects of adenosine signaling in the development of cancer vaccines.

## 2 Local adenosine generation, its dynamic regulation, and metabolism

**Figure 1:**
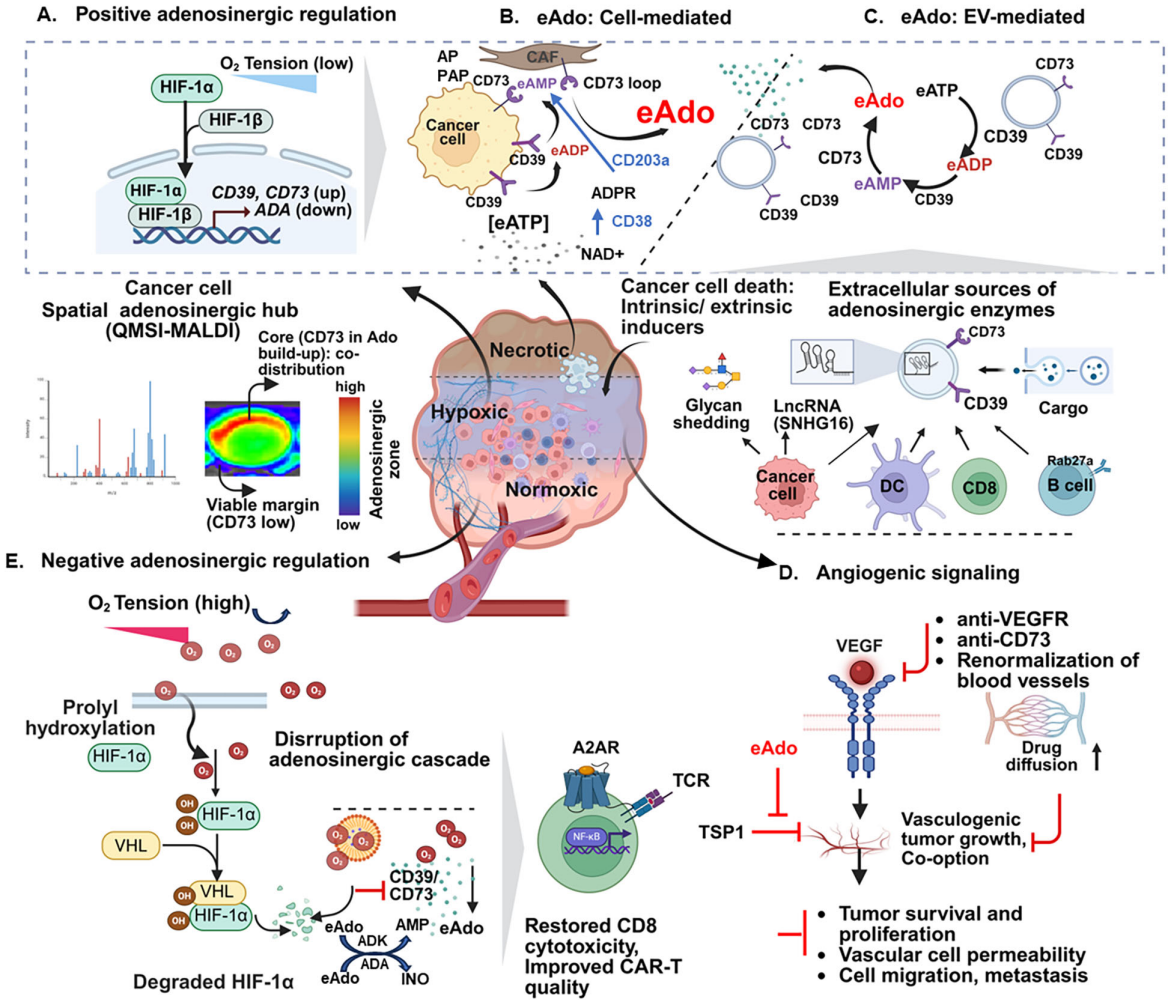
Key factors that orchestrate adenosine production in the tumor microenvironment. Three physiologically distinct layers —a viable, vasculature-rich outer layer, a hypoxic middle layer, and a necrotic inner layer — form a continuum to propel Ado generation in the TME. Adenosinergic regulation is mediated by HIF-1α/HIF-1β signaling, leading to CD39 and CD73 transcriptional upregulation **(A)**. CD39 and CD73, expressed on the surface of cancer and stromal (CAF) cells, are vital sources of ectoenzymes. CD39 and CD73, expressed on EVs, also complement adenosinergic signaling **(B, C)**. Generation of eAdo involves the conversion of eATP to eADP and eAMP by CD39, followed by the final conversion of these eAMP into eAdo. Non-canonical conversion of NAD+ to ADPR and AMP is catalyzed by CD38 and CD203a (ENPP1). Quantitative mass spectrometry imaging (qMSI-MALDI) and spatial metabolomics reveal the relative abundance of purinergic enzymes and purines, including adenosine, across different regions of the tumor. Hypoxia and Ado also trigger angiogenic blood vessel formation and promote VEGF-mediated events. Anti-VEGFR, anti-CD73, and renormalized blood vessels facilitate the diffusion of drugs to tumors. A red arrow with a blunted end indicates key inhibition points **(D)**. In the normoxic region, the degradation of HIF-1α by prolyl hydroxylases and VHL limits the expression of CD39/CD73 and Ado but supports ADK and ADA-mediated metabolism of Ado, thereby regulating context-dependent CD8 activation. Oxygen-carrying agents can attenuate Ado generation and avert immune suppression **(E)**. QMSI, Quantitative Mass Spectrometry Imaging; MALDI, Matrix Assisted Laser Desorption/Ionization; HIF-1,Hypoxia-Inducible Factor 1; ADA, Adenosine Deaminase; ADK, Adenosine Kinase; NAD^+^, Nicotinamide adenine dinucleotide; ADPR, Adenosine diphosphate ribose (ADPR), AP, Alkaline Phosphatase; PAP, Prostatic Acid Phosphatase; CAF, Cancer Associated Fibroblasts; EVs, Extracellular Vesicles; VHL, Von Hippel-Lindau; VEGF, Vascular Endothelial Growth Factor; TSP1,Thrombospondin-1. Image created using BioRender.com.

The intra-tumoral adenosine gradient regulates purinergic signaling in the tumor microenvironment (TME). Central to this gradient are a) dying tumor cells resulting from exposure to various external and internal stressors like nutrients and oxygen deprivation, as well as therapy-induced tumor death; and b) a hypoxic middle tumor layer rich in ectoenzymes, along with the outer invasive tumor margin mainly containing viable tumor cells (24, 25) (**Figures 1A–C**). Each layer contains immune cells with varying plasticity and signaling deregulation. The proximity of these cells to the local extracellular adenosine (eAdo) gradient and other metabolites dictates their dysfunction. Therefore, profiling the spatially polarized Ado gradient in tumor-proximal compartments is pivotal for evaluating its impact on prognosis and therapeutic responses (26–28). Given the physical instability of Ado, efforts are ongoing to capture its dynamics, prognostic value, and reliable surrogates in both time and space (29–31). The production of eAdo is regulated by two ectoenzymes: CD39, expressed on epithelia, endothelia, and specific immune cells, converts eATP first into eADP and then into eAMP. A second enzyme, ecto-5’-nucleotidase (i.e., CD73), rapidly converts eAMP into eAdo. Both these enzymes are more active in hypoxic regions, increasing eAdo concentration from 30–200 nM to 30 μM (32–35). In the non-canonical pathway, tissue-nonspecific alkaline phosphatase (TNAP), as well as other enzymes such as CD38, ectonucleotide pyrophosphatase/phosphodiesterase 1(ENPP1)/CD203a, and prostatic acid phosphatase (PAP), can convert NAD+ into eAdo (8, 36, 37) (**Figure 1B**). The adenosine ‘halo’ refers to a space encircling outside immune cells that is decorated with adenosine, and visible mainly in CD39/CD73-rich hypoxic TME. These hotspots are integral parts of the adenosinergic engine (38). Technical advances (MALDI-qMSI) in spatially resolved quantitative mass spectrometry imaging further illuminated this feature (39). This spatial framework integrates single-cell protein and metabolite profiling of immune and cancer cells (40). High CD73 mRNA expression is associated with the progression of GBM. A spatially resolved pathology and MSI footprint revealed regions with high CD73 expression, which are correlated with elevated adenosine levels (26) (**Figure 1A**). The upstream events in adenosine generation, hypoxia dependent expression and function of adenosinergic enzymes, and control of negative regulators are presented in **Figure 1A**.

### 2.1 The adenosinergic network is regulated by the hypoxia axis

HIF-1α, stabilized under low oxygen conditions, triggers transcription of CD39 and CD73. Prolyl hydroxylase enzymes (PHDs) hydroxylate HIF-1α before its degradation by the proteasomal pathway (41, 42). The von Hippel-Lindau (VHL) targets HIF-1α and HIF-2α for degradation, whose mutations can lead to stabilization of HIFs even when oxygen levels are normal (43). Production of adenosinergic enzymes is disrupted as a result of HIF-1α degradation. Turning off the adenosinergic network has direct consequences on the recovery of tumor-reactive T cell functions, which tend to avoid hypoxic regions in the TIME (21, 22). Hypoxia-modulating drugs targeting the CD39-CD73-A2AR axis reinvigorate immune cells (44–46). CD73, under hypoxia, promotes angiogenesis. Combined blockade of CD73 and bispecific PD-1-VEGF antibody can reverse tumor growth inhibition (47). A2AR can promote angiogenesis by inhibiting thrombospondin1 (TSP1) (48). Its upregulation correlates with poor response to anti-vascular agents and limits survival (49). Normalization of tumor blood vessel function can facilitate drug diffusion and slow tumor growth (50, 51). Besides angiogenesis, vessel co-option can enable tumor growth and metastasis by utilizing existing tumor vasculature (52, 53) (**Figures 1D, E**).

### 2.2 CAF as a source of CD73 in propelling adenosine generation

The stromal context of adenosine receptors (ADOR) is a critical determinant of their network complexity. In CRC, cancer-associated fibroblasts (CAF) spearhead an A2BR-mediated feedforward activation loop by elevating surface CD73, which further facilitates a massive release of eAdo and ultimately blunts TILs activation in a mechanistic cascade. Notably, the MC38-derived syngeneic CRC model is CAF-deficient; therefore, CD73 targeting in the CAF compartment is not efficacious in this model (25). In human breast cancer (BC), CAF-S1 drives recruitment and subsequent differentiation of CD25+FOXP3+ regulatory T cells (Treg) through the engagement of surface B7H3, DPP4, and CD73 signaling. This phenotype finally impairs T cell effector function (54). These studies bring forward the adenosinergic roles of the stromal compartment.

### 2.3 CD39 and CD73-expressing exosomes complement adenosinergic signaling

Tumor-derived circulating exosomes induce resistance to immune checkpoint blockade (ICB) therapy in lung cancer and melanoma (55, 56). Similar roles of exosomes in the adenosinergic pathway have been demonstrated. For instance, activated CD8 cells can release CD73-containing extracellular vesicles (EVs). These EV-bound CD73s contribute to adenosine production (57). EVs facilitate intercellular transfer of lncRNA SNHG16 and the subsequent development of an adenosine-producing CD73+ γδ1 Treg subset (58). Cancer-derived exosomes enriched in CD73 and CD39 suppress T-cell responses through complementary adenosinergic signaling (59). As discussed later (section 4.4.4, 147), B cell-specific Rab27a mediates the release of EVs that carry CD39 and CD73 as cargo (60). Dendritic cell (DC) and tumor-derived EVs can induce extracellular adenosine (eAdo) production by Tregs using CD39 and CD73 (61–63). Additionally, the tumor cell membrane sheds glycan. Its uptake by Tregs upregulates adenosinergic conductors CD39 and CD73 (64) (**Figure 1C**).

### 2.4 ENT1, ADK, and ADA in regulating adenosine signaling

**Figure 2:**
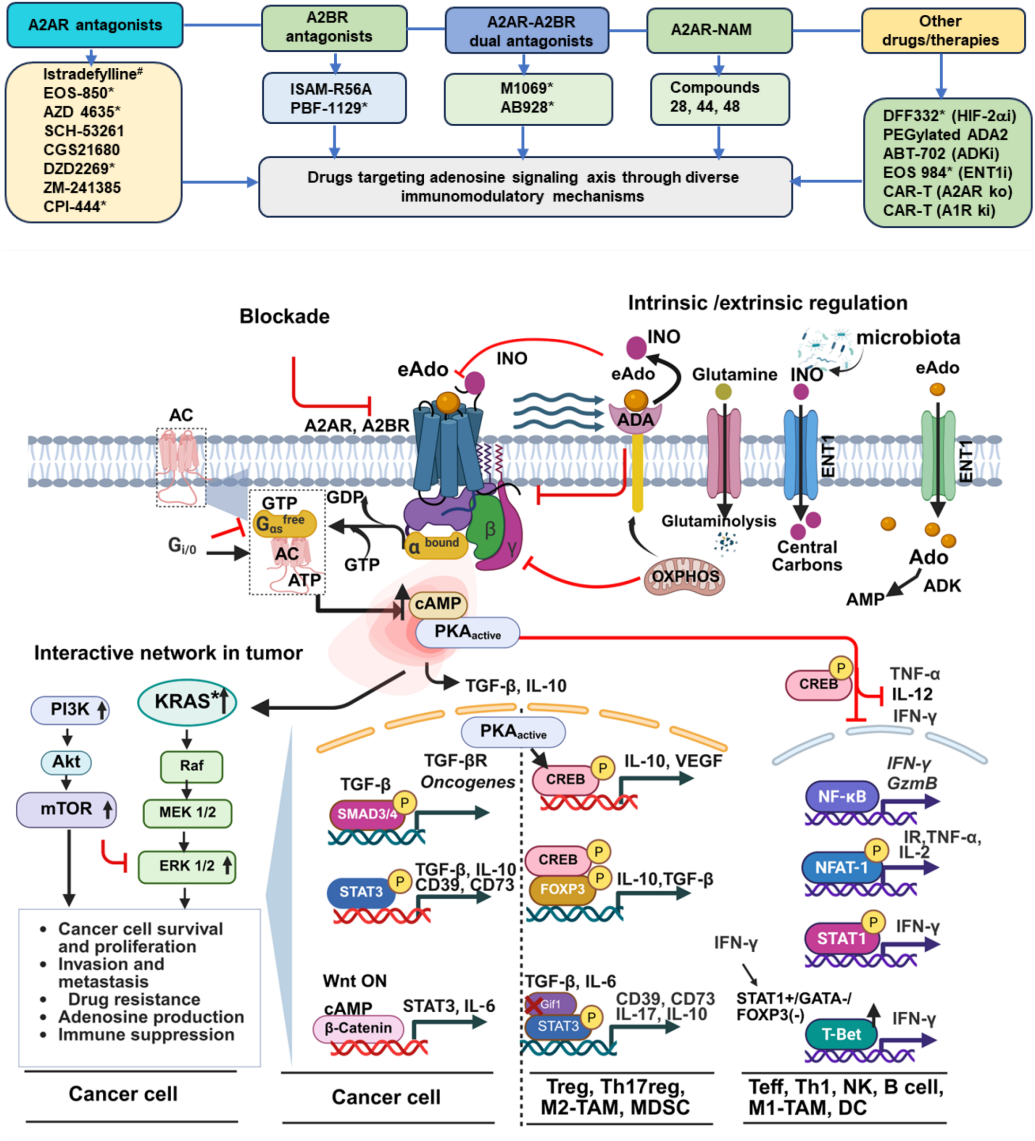
Adenosine receptor signaling and its regulatory networks converging tumor and immune cells. Drugs targeting various aspects of adenosine receptor signaling (top). The binding of Ado to A2AR (high affinity) and A2BR (low affinity) in tumors initiates divergent signaling in both cancer cells and immune cells. Liganded receptor targets a heterotrimeric G protein complex (Gαβγ). The subsequent dissociation of Gα triggers Gαs-mediated activation of the AC and cAMP-PKA complex. Signal emanates from Gi/o interferes with Gαs-AC interaction. The cAMP-PKA complex finally exerts its effects via pCREB. In cancer cells, it initiates downstream oncogenic signaling cascades such as i) PI3K/Akt/mTOR, ii) KRAS-Raf-ERK1/2, iii) TGF-β-SMAD3/4, iv) STAT3, and v) Wnt-β-catenin. In immune cells, the cAMP-PKA-mediated pCREB axis impairs NF-κB, NFAT-1, STAT1, and T-Bet to suppress pro-inflammatory responses in effector cells. CREB cooperates with FOXP3, SMAD3/4, and supports Tregs, Th17reg, M2-TAM, and MDSC via TGF-β and IL-10 loops. Several cooperative and counterregulatory molecules also regulate Ado level. ADA depletes Ado by converting it to INO. ADA action following glutamine uptake and breakdown, and OXPHOS accumulates INO that competes with Ado. The microbiome can serve as an extrinsic source of INO. INO serves as an alternative carbon source for supporting CD8 cells. The accumulation of Ado through ENT1 can reverse Ado to AMP by ADK, and contextually inhibit CD8 function. Inhibition caused by therapy or intrinsic or extrinsic factors at a particular step is indicated by a red line with a bent end (bottom). *Candidates in oncology clinical trials. ^#,^Approved for PD. GEF, Guanine Nucleotide Exchange Factor; AC, Adenylate Cyclase; PKA, Protein Kinase A; CREB, cAMP Response Element Binding protein; PTEN, Phosphatase and TENs in homolog deleted on chromosome 10; NFAT-1, Nuclear Factor of Activated T-cells; T-bet, T-box expressed in T cells; Gif-1, GRF-Interacting Factor, SMAD, Suppressor of Mothers against Decapentaplegic; INO, Inosine; OXPHOS, Oxidative Phosphorylation; ADA, Adenosine Deaminase; ENT1, Equilibrative Nucleoside Transporter 1; ADK, Adenosine Kinase; NAMs, Negative Allosteric Modulators; CAR-T, Chimeric Antigen Receptor-T, A2AR ko, A2AR knock out, A1R-ki, A1R knock in; Bottom image created using BioRender.com.

The physiological eAdo level is maintained by equilibrated nucleoside transporter 1 (ENT1) and intracellular adenosine kinase (ADK) (24, 65). Once inside the cells, Ado has two possible fates: a) it is either phosphorylated by ADK to AMP, or b) it is converted to inosine (INO) by adenosine deaminase (ADA). Their repressive state in hypoxia protects Ado (24, 32). Likewise, ENT1 facilitates Ado uptake in activated CD8 cells, blocking their expansion. ADK inhibition restores T cell proliferation in environments with high Ado, which can also impair pyrimidine biosynthesis in T cells (39). Additionally, Ado levels can be reduced by ADA. Its isoform (ADA2) is active at acidic pH (66). ADA2 inhibits tumor growth by depleting Ado (67). The stable but low-affinity INO hinders the binding of Ado to its cognate receptors and thereby inhibits downstream cAMP-PKA but displays overall bias to pERK1/2 signaling (68–70). Glutamine uptake, glutaminolysis, and OXPHOS increase the expression of INO through ADA. This INO uncouples eAdo signaling from A2AR and rescues CD8 cell-specific NFκB signaling (71). INO is an alternative carbon source to support CD8 cells (72). INO, derived from the microbiome, can boost ICB response in an A2AR-proficient environment (73), indicating critical competitive roles of this metabolite (**Figure 2**).

## 3 Adenosine receptor subtypes and their druggability

### 3.1 Druggability of A2AR and A2BR in cancers

Structure-based drug design has recently shed light on the roles of A2AR (74, 75). The ubiquity of A2AR poses hurdles for subtype-selective antagonists and potential on-target off-tumor effects in normal tissues (8). The pharmacokinetics (PK) of A2AR and A2BR antagonists have been evaluated for their linearity in relation to pharmacodynamics (PD). The poor PK profile of antagonists in the past has led to trial failure (76). Istradefylline is the first A2AR antagonist approved for the treatment of Parkinson’s disease (11). Imaradenant (AZD4635) is the first A2AR inhibitor developed as a cancer immunotherapy agent. It mainly functions by relieving suppression on the tumor microenvironment (TME) following engagement of eAdo (77). A2AR has a hydrophobic sub-pocket, and antagonist binds to the receptor, blocking its ribose binding region. When antagonists bind at the orthosteric site, they induce a conformational change (inactive), keeping this pocket unoccupied (78). Second-generation antagonists such as ciforadenant, etrumadenant (AB928), imaradenant (AZD4635), and inupadenant (EOS-850) showed high affinity and potency for A2AR antagonism (Ki <5 nM in all cases). AB928 has Kd values of 1.4 nM and 2 nM for A2AR and A2BR, respectively (30). The PK profile of AB928 exhibits a clearly linear pattern, proportional to the doses. It is supported by marked inhibition of agonist-induced pCREB at peak plasma concentrations across all doses. At plasma levels ≥1 μM, it results in ≥90% target inhibition (30). Elevated adenosine levels are found in the TME rather than the brain. There is a need to design A2AR antagonists that are efficacious at low doses and at the same time peripherally restricted, thereby avoiding central nervous system (CNS) penetration. For example, inupadenant is a non–brain penetrating antagonist, offering a preferable safety profile for systemic oncology applications (AACR, 2020, Abstract nr CT152). In contrast, istradefylline (Ki of 12 nM for A2AR) is effective for Parkinson’s disease due to CNS penetration (79). Its derivatives have been developed to improve the photostability (80). Another A2AR antagonist, ANR 672, aiming to target GBM, showed moderate CNS permeability (81). Some of these and other agents covered in this article are schematically presented in **Figure 2**. To combat dose-related issues, aberrant selectivity, and toxicity, A2AR-directed non-competitive negative allosteric modulators (NAMs) have been developed. NAMs (compound 28,44,48) inhibit through a conformational change of the receptor, even in a high Ado environment. They are superior in avoiding activity loss and restoring T cell immunomodulation in high (5 μM) NECA conditions compared to lead orthosteric candidates (82).

### 3.2 A2AR and A2BR in the proximal cAMP signaling

Second messenger cAMP plays a key role in the A2AR and A2BR signaling pathways (83). Understanding the dynamics and divergence of cAMP in the ADOR pathway is crucial for pharmacodynamic profiling of drugs targeting this network. Membrane-bound adenylate cyclase (mAC), designated as AC 1-9, converts ATP into cAMP through its interaction with G proteins. In contrast, AC10 is a cytoplasmic form and remains uncoupled from G proteins (84). Upon eAdo binding to A2AR or A2BR, adenylate cyclase (AC) gets activated. This regulatory switch is controlled by distinct G protein subunits (Gαs or Gαo/i), which bind to AC. Ado binding to its receptors acts as a guanine nucleotide exchange factor (GEF), releasing Gαs from its heterotrimeric form in the cytosol (85). The activation of AC then produces cAMP from intracellular ATP. Conversely, when Ado binds to A1R, Gαo/i interacts with AC to compete with Gαs, inhibiting AC activity and lowering intracellular cAMP levels (86). Elevated cAMP levels activate PKA, which then translocates to the nucleus to phosphorylate the transcription factor CREB (87, 88). A “buffered diffusion model” suggests that cAMP diffuses rapidly to distant binding sites, such as protein kinase A (PKA), when binding sites on GPCRs are saturated due to high agonist concentrations (89). This model offers key insights into how cAMP distribution varies under conditions of heterogeneous ligand availability. Finally, activation of the cAMP signaling pathway promotes proliferation and suppresses apoptosis in cancer cells (90) and suppresses pro-inflammatory signaling (**Figure 2**).

### 3.3 Spatial CD73-adenosine context in co-targeting CD73-A2AR

In triple-negative breast cancer (TNBC), a lower level of CD73 is more predictive of response when compared with TILs in the stromal compartment (91). A study by Graziano et al. revealed that in PDAC, M2-polarized A2AR-high tumor-associated macrophages (M2-TAMs) coexist with heterogeneous but eAdo-rich necrotic and hypoxic regions in the TME. Co-targeting CD73 and A2AR is a potential therapeutic approach to combat this challenge. In ICB-resistant cancer, M2-macrophages express high A2AR. Concurrent inhibition of CD73 and A2AR remodels the TME by impairing the recruitment of M2-TAMs and Treg cells. It reduces tumor growth and the burden of metastasis (31). Several innovative oxygen supplementing platforms have shown promising outcomes in decreasing CD39/CD73 and eAdo levels in TME by directly attenuating hypoxia (44–46).

## 4 Mechanistically versatile adenosine signaling and TIME

The presence of ADOR subtypes in all key immune subsets underscores the potential for targeting multiple pro- and anti-inflammatory immune hubs to counter tumor evasion (outlined in **Figure 2**). A systematic understanding of this complex network and its perturbation is crucial for deciphering the crosstalk and identifying vulnerabilities.

### 4.1 Protective roles of adenosine signaling in cancer cells

Adenosine signaling communicates with its autocrine-paracrine interactive network, promoting tumor growth and metastasis. TGF-β, an output of this autocrine-paracrine loop, can interact directly with PKA, promoting SMAD4 and pCREB-mediated mesenchymal phenotypes and tumor invasion (92, 93). A2AR signaling crosstalk with PI3K-AKT-mTOR, as well as aberrant signaling in the Wnt/β-catenin pathway in tumor cells, drives tumor progression and metastasis (94–96). Han et al. demonstrated that, in EGFR mutant ALK-positive NSCLC, which is nonresponsive to PD-1 inhibitor, the upregulation of CD73 via the ERK-Jun pathway amplifies CD73 at the genomic level (97). KRAS, known for its roles in immune evasion, can mechanistically engage the CD73-A2AR signaling axis in this process (97, 98). Momentum is gaining in developing drugs against clinically prevalent oncogenic KRAS mutant cancers (99, 100) (**Figure 2**).

Drugs perturbing tumor intrinsic upstream signaling of adenosine receptors usually exhibit a paucity of direct effects on tumor killing at low doses. A GBM-directed anti-A2AR agent, istradefylline (IST), showed an inferior anti-proliferative effect on cancer cells compared to riluzole, which mainly inhibits a downstream player, Casein kinase I delta (CK1δ) (101). IST restricts malignancy-promoting properties and potentiates the effect of paclitaxel on melanoma cells *in vitro*. At the same time, it induces a compensatory loop via ADA loss to generate adenosine (102). These data suggest that while cancer cell-specific adenosine signaling is crucial for its survival, proliferation, invasion, and metastasis through its crosstalk within the oncogenic network (**Figure 2**), its primary function is to generate adenosine. Targeting A2AR and A2BR in cancer cells within immunodeficient contexts exerts limited anti-tumor effects, reaffirming their credentials as metabolic immune suppressors.

### 4.2 A2AR and A2BR signaling in regulatory and suppressor immune cells

Cytokines such as IL-10, VEGF, and TGF-β are products of adenosine signaling. They are central to immunosuppressive and oncogenic transcription programs (103). They act as conduits for pCREB, canonical SMAD3, or SMAD4 (92, 93). For example, using a regulatory loop, STAT3-driven expression of CD39 and CD73 in tumors elevates Ado (104). Additionally, A2AR, FOXP3 and STAT3 regulate various regulatory and anti-inflammatory functions in Treg, M2-TAM, and myeloid-derived suppressor cells (MDSC) (105, 106) (**Figure 2**). A2AR and A2BR, through activating PKA and exchange protein directly activated by cAMP (Epac), also polarize DCs to a suppressive phenotype, blunting NF-κB activity and IL-12 production (107). The cAMP-PKA-pCREB axis further represses IRF-4 and KLF-4, and at high cAMP levels, reprograms cDC2s to induce Th17 polarization and bias (108). Similarly, suppression of Gif1 by TGF-β, coupled with IL-6, enables STAT3 to upregulate the CD39 and CD73 genes in Th17reg cells (109).

### 4.3 A2AR and A2BR signaling negatively modulate effector and helper cells

The downstream effects of A2AR and A2BR on TCR and myeloid signaling polarize M1-M2 macrophages, instructing the suppression of effectors and a shift from Th1 to Th2 phenotypes (103). In the immune compartment, cAMP inhibits CTL activation through disrupting the TCR signaling pathway (110). The cAMP/PKA axis suppresses the production of IL-12, IL-2, TNF-α, and IFN-γ, as well as their regulatory circuits, which operate through the NF-κB and NFAT pathways. Molecularly, pCREB and its downstream cascade are key regulators of suppressing cytotoxic TILs. The A2AR/PKA/mTOR pathway inhibitors can reverse these effects (111). Nuclear pCREB acts as a negative regulator of NF-κB in effector T-cells and as a positive regulator of FOXP3 in Tregs (112, 113). Notably, NFAT1 is found to be suppressed by PKA using a specific cAMP analogue (114). A cAMP-dependent nuclear localization of inducible cAMP early repressor (ICER)/cAMP response element modulator (CREM) in natural regulatory (nReg) T cells represses NFATc1 in conventional T cells, leading to the suppression of IL-2 production (115). Mechanistically, Th1 cytokines, such as IFN-γ, can activate STAT1 and T-bet in an autocrine-paracrine loop, while inhibiting GATA3 and thereby restraining Th2 polarization. PKA-driven cytokines, such as IL-4, can skew this lineage in favor of Th2 (116, 117). In conclusion, the dual roles of the cAMP-PKA axis are context-driven. The two-pronged strategy adopted by the adenosinergic and A2AR/A2BR axis therefore steers the differential regulation of pro- and anti-inflammatory signaling. Additionally, the heterogeneity in A2AR and A2BR signaling suggests that one or more transcription factors cooperate at the promoter/enhancer levels or repress gene expression programs in various immune subsets (**Figure 2**).

### 4.4 Key immune cell types in adenosine signaling modulation

An elaborate ADOR signaling in the immune microenvironment suppresses both innate and adaptive immune responses and has emerged as a significant barrier to available therapies (outlined in **Figure 2**). There have been several excellent reviews in recent years that discussed the main protagonists in both innate and adaptive networks, as well as their complex interplay in cancers (118–120). Here, we primarily discuss the recent advances in understanding the A2AR and A2BR, as well as their roles in regulating the functional phenotypes of various immune subsets.

#### 4.4.1 A2A and A2B adenosine receptors in cytotoxic CD8 cell dysfunction

**Figure 3:**
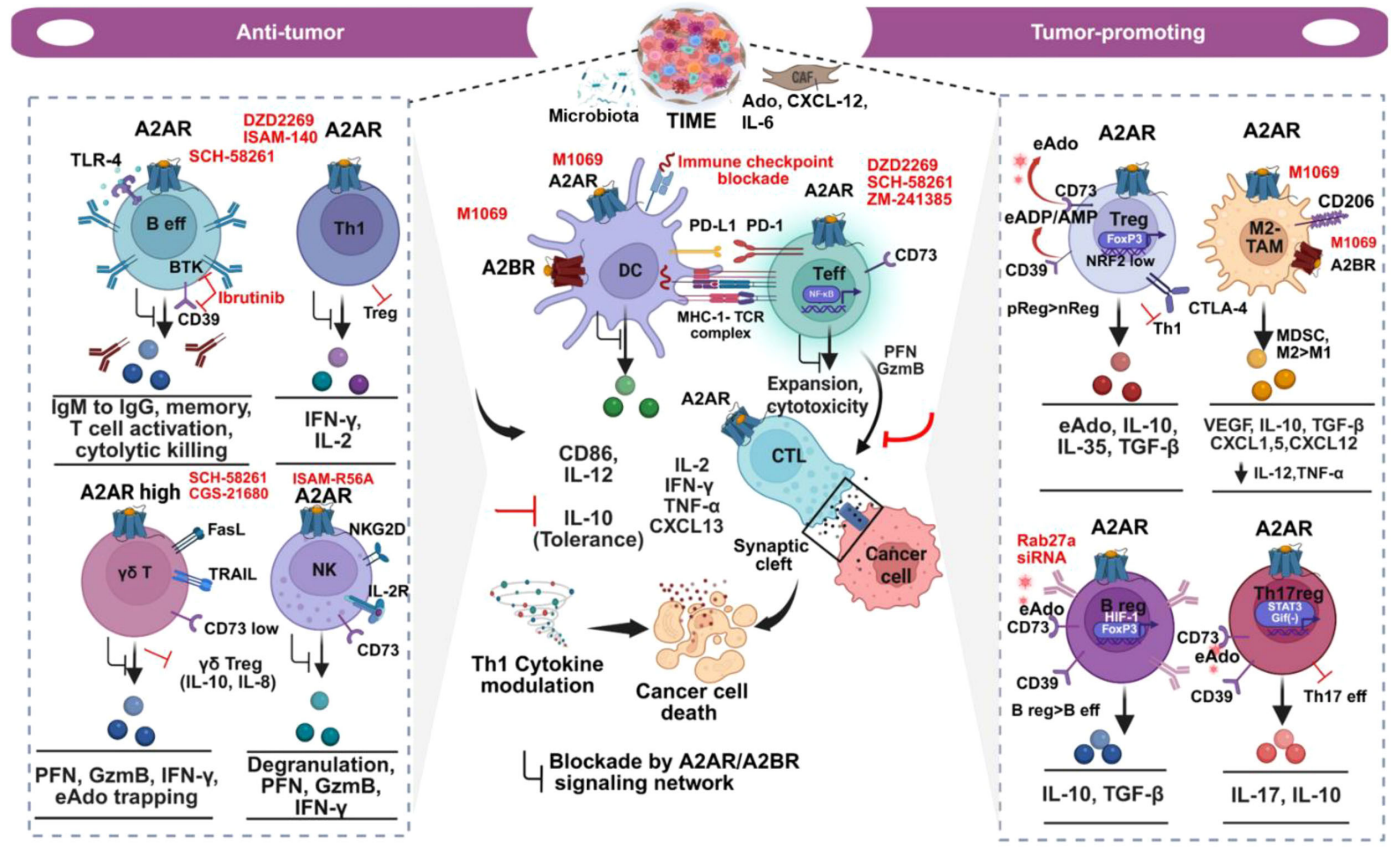
A2AR and A2BR panorama in pro- and anti-tumor mechanistic immune hubs. Through engagements with high-affinity A2AR or A2AR and A2BR collaboration, eAdo impairs pro-inflammatory signals in B effector, T helper, NK, and γδT cells, dampens their cytokine/cytotoxic functions, and alters their phenotypic identity. Ibrutinib mimics Ado effects in B-eff, but in negative feedback, it can suppress CD39 (right). Primarily, eAdo engages with low-affinity A2BR in myeloid cells. Dual action A2AR-A2BR antagonists reverse this suppression. The same A2AR and A2BR promote regulatory immune cells (Treg, Breg, Th17reg) and MDSCs/M2-TAM, leading to their differentiation and anti-inflammatory and tumor-promoting functions (right). A2AR and A2AR or A2BR dual antagonists relieve depressed DC from Ado influence, reinvigorate effector T cells, stabilize cytolytic T cells-cancer cell synapses, restore CTL expansion, and avoid tolerance (centre). Phenotypic plasticity and shift in Th1 to Treg, γδT to γδTreg, nReg/pReg, M1 to M2-paradigm (TAM), Th17 to Th17reg, and Beff to Breg tilt the dynamics. A black arrow with a blunt end indicates the downstream inhibitory effects of A2AR signaling (left). The red arrow with a blunt end indicates interventions caused by a particular therapy or impact of the autocrine-paracrine inhibitory loop at a specific step (left, right). TLR4, Toll-like Receptor 4; BTK, Bruton Tyrosine Kinase; TRAIL, TNF-Related Apoptosis-Inducing Ligand; nReg, natural Regulatory cells; pReg, peripheral Regulatory cells; NRF2, Nuclear factor erythroid 2-related factor 2. Image created using BioRender.com.

CD8 cells are subject to direct and indirect dysregulation following engagement of A2AR and A2BR (outlined in **Figure 3**). Proximity ligation assay showed that an A2BR heterodimer with A2AR reduces the ligand affinity for A2AR compared to a high-affinity A2AR homodimer (121). Interestingly, A2AR and A2BR demonstrated the ability to undergo homo- and heterodimerization in immune cells. In patient-derived BC spheroids, A2BR inhibition by its selective small-molecule inhibitors (SMI), such as ISAM-R56A, elicited T-cell and NK cell-mediated anti-tumor responses when these cells were co-cultured with tumor cells. Notably, dual A2AR/A2BR or independent inhibition of A2BR delivers superior anti-tumor responses than an A2AR antagonist alone. A sub-apoptotic dose of the A2BR antagonist ISAM–140 was found to rescue naïve and effector CD8, as well as the central/effector memory CD4 cells. Similarly, ISAM-R56A recovered the NK cell function and, to some extent, T cell proliferation. Both agents at intermediate doses showed modest killing of tumors in the absence of immune cells. Notably, flow cytometry data revealed no remarkable differences in the percentage of A2AR and A2BR-positive populations in the lymphocyte subsets (11.2%) from healthy donors (122). A2BR deficiency or pharmacological A2BR blockade improves T cell-priming by DC and adoptive T-cell transfer (ACT), suggesting that A2BR exerts its effects on T cells mainly through myeloid cells (123). A highly selective dual A2AR and A2BR antagonist, M1069, in an Ado high environment, restored T cell-mediated IL-2 production. M1069 suppressed tumor-promoting chemokines, such as CXCL1 and CXCL5, and the myeloid-driven VEGF. Moreover, in response to M1069, DCs primed with Ado were found to recover from depressed IL-12 production and regain their T-cell-stimulating properties. In BC-derived mice 4T1 cell line expressing high CD73 and Ado, this dual antagonist synergized with bifunctional fusion agent of anti-PD-L1-TGFβ (bintrafusp alfa or BA) or platinum agent *in vivo* (124). These findings offer interesting insights into A2AR- and A2BR-driven combinatorial therapy, particularly given that BA alone could not achieve superior efficacy in the initial trial (125). Several strategies targeting the adenosinergic pathway have been evaluated for their anti-tumor effects. For example, a humanized anti-CD73 antibody acting through CD73 internalization relieves the checkpoint in CD11b+granzyme-high cells, allowing for their expansion. In contrast, A2AR knockout (KO) in mice endorses CTL expansion; at the same time, it increases the expression of CD73. Tumors use this feedback loop as an evasive strategy to counterbalance the lack of A2AR signaling. The CD73 and A2AR co-blockade exerts more efficient tumor growth inhibition and anti-invasive or anti-metastatic responses than single blockade (126). Mechanistically, the knockout of A2AR triggers CD8 infiltration and increases the number of CTLs in tumor-draining lymph nodes (TDLN). It further delays the growth of lymphoma and reduces the lung colonization of tumor cells (127). These observations highlight the existence of nonredundant loops in A2AR signaling (126, 127). Selective antagonism of immune-specific A2AR boosts the stabilization of tumor-immune synapses critical for delivering tumor-targeted lethal hits. Further dissection of this structure revealed that the engagement of A2AR by eAdo impairs the polarization of cytolytic granules at immune synapses and disrupts the micro-cluster density of pSrc during TCR signaling in CTL. ZM-241385, a potent A2AR-selective antagonist, reverses this standoff (128) (**Figure 3**). Similarly, PD-L1 blockade, when combined with inhibitors of CD73 and A2AR, yields an anti-tumor response. Whole exome, targeted deep sequencing (TDS), RNA seq., and spatial biology profiling in patient-derived tissues of diffuse large B-cell lymphoma (DLBCL) identified a grade 1 dysfunctional CD8 fraction. This fraction is defined by PD-1+CD8 or A2AR+CD8 phenotypes and is associated with poor OS. A grade 2 dysfunctional CD8, defined by PD-1+A2AR+CD8 subset, showed the worst PFS and OS. The study also deciphered that loss-of-function CD73 gene mutations and deletions can result in better patient survival compared to their CD73 wild-type counterparts (129). Like small molecule inhibitors (SMI), protein degraders also raised optimism in targeting the immune checkpoints. For example, tripartite motif-containing protein 21(TRIM21) functions as a ubiquitin ligase that directs proteasomal degradation of CD73 in tumor cells. By reducing the tumor-derived eAdo levels, TRIM21 boosts CD8 activation. Altered amino acid residues in the CD73 structure or deubiquitylation of CD73 by OTU domain-containing protein 4 (OTUD4) disrupt its interaction with TRIM21. Subsequent accumulation of eAdo causes suppression of CD4 and CD8 functions (130, 131).

#### 4.4.2 TGF-β-Th17-adenosinergic signaling

Depending on the combinations of cytokines they are exposed to, Th17 cells can assume either a regulatory (Th17reg) or an effector (Th17eff) phenotype (132, 133). The link between these two subsets and adenosinergic signaling, however, is yet to be fully understood. Independent studies have shown either neutral or positive involvement of TGF-β as a molecular switch in the emergence of inflammation-associated pathogenic Th17 phenotypes (134–136). Th17 reg can release Ado, allowing it to suppress Th1 and Teff functions. Th17 cells express high levels of CD39 and CD73 through IL-6-mediated STAT3 activation. TGF-β in these cells downregulates Gif1, a transcriptional repressor of CD39 and CD73 (109) (**Figure 3**).

#### 4.4.3 Unique roles of Tregs in promoting adenosine signaling

A2AR signaling directly guides the phenotypic development of Tregs, which have definitive roles in impairing effector cells (105). Thymic-derived Tregs are natural Tregs (nTregs), whereas peripheral Tregs (pTregs) are developmentally induced. Tan SN et al. recently reported substantial presence of these pTregs in the TME, which are terminally differentiated from IFN-γ+Th1 cells. Tumor-derived TGF-β converts Th1 cells to pTregs. These tumor-resident pTregs, like their precursor Th1 cells, depend on T-bet and display high cell-surface CD39, which can enable strong paracrine Ado signaling in CD8 cells to suppress their effector function (137). Another enigma of Tregs is that these cells are vulnerable to oxidative stress due to the sheltering of an inherently weak nuclear factor erythroid 2-related factor 2 (NRF2). Consequently, these cells succumb to high oxidative stress induced by free oxygen radicals. The subsequent rise of eATP by the apoptotic Tregs, along with their high expression of CD39 and CD73, converts this eATP to eAdo and perturbs the anti-PD-L1 blockade. Interestingly, the observed effect in this case occurs without contributions from Treg cytokines, such as TGF-β, IL-10, and IL-35, or checkpoints like PD-1 and CTLA-4 (138) (**Figure 3**). Radiation-induced infiltration of CD4 and CD8 cells, in cooperation with A2AR blockade (DZD2269), reduced Treg recruitment. An increase in cytotoxic T cell functions under this combination rescued the anti-tumor responses (139).

#### 4.4.4 Emerging roles of B cells in adenosine signaling

Research over the past few decades has focused extensively on the biology of TILs. Notably, after the discovery of tertiary lymphoid structure (TLS) as a specialized microenvironment niche by Picker, L. J. & Butcher, E. in 1992 (140), in recent years, there has been a paradigm shift from the alleged tumor-promoting roles of B cells to their association with improved prognosis and clinical outcomes (141–144). However, there is a paucity of data underpinning the roles of B cells in A2AR and A2BR signaling in cancers. Experimental evidence suggests an interplay between the hypoxia and adenosinergic axes in shaping a restrained B cell niche in the germinal center (GC). This results in CD73 upregulation and an A2AR-induced reduction in B cell and Tfh numbers. A concomitant increase in follicular regulatory T cells (Tfr) primes this suppressive milieu (145). Interestingly, like Treg, CD73+Breg produces eAdo using its surface CD39 and CD73 (schematized in **Figure 3**). However, eAdo binds to A2AR only in CD73-negative B-eff, showing a distinct signaling bias. Through inhibiting Bruton Tyrosine Kinase (BTK) signaling and blocking Ca^2+^ influx, Breg-derived eAdo can suppress the B-eff function in the tumor. While BTK inhibitor ibrutinib mimics these effects, by utilizing a negative feedback mechanism, it downregulates CD39 level and reduces eAdo accumulation. A2AR inhibitor reduces tumor growth and absolute TILs counts *in vivo* (146). Other data showed that Rab27a promotes B-cell-mediated EV secretion. Upon exposure to therapy, B-cell-derived EVs expressing CD39 and CD73 generate Ado, thereby engaging the A2AR in T cells. Rab27a gene transcription bolsters EV accumulation in low-oxygen conditions. Silencing of the Rab27a gene attenuates the suppressive effects of adenosinergic EVs and improves chemotherapy responses in humanized xenograft mice (60, 147). B cells carve a niche in cancer immunotherapies (148). CD39 on CTL expresses chemokines such as CXCL13, which is required for recruiting CXCR5+B cells in TLS, but higher CD39 and CD73 expression in stroma through increased Ado level creates a barrier for these cells to reach the tumor nest (149). Future studies will focus on the involvement of adenosinergic network in disrupting TLS, whose neogenesis is likely to redefine the roles of B cell-guided modalities as reported recently (ESMO-IO Congress 2024, 174MO, https//doi.org/10.1016/j.iotech.2024.100927). AT-1965 is an investigational new drug with B-cell stimulating properties that is currently in a Phase 1/2 F-I-H clinical trial for multiple advanced solid malignancies (NCT06234098).

#### 4.4.5 NK and γδ T cells dysfunction in eAdo microenvironment

Unlike CD8+ effector T cells, NK cells lack endogenous TCR. By fine-tuning signals of activating receptors, such as NKG2D, they recognize tumor cells and exert their cytotoxic effects when MHC-1 is lacking. This is important given that MHC-1 deficit prevents the engagement of NK inhibitory receptors (150). Activated NK cells in the blood enable the killing of circulating tumor cells that escape the slowly evolving CTL surveillance (151). NK cells-derived chemokines, such as CCL5, CXCL1, and CXCL2, are crucial for recruiting conventional DC1 (cDC1) to tumor sites and the subsequent activation of T cells (152). These advantages inspired the recent development of CAR-NK, including CAR γδ T cells (153–155). However, unlike in T cells, the roles of A2AR in NK cells are less documented (156). A2AR and A2BR antagonists suppress the metastasis of CD73-positive BC and melanoma, but only A2AR blockade or deletion recovers NK cell maturation and cytolytic effects (157). NK cells express A2AR, and tumor-infiltrating NK cells express higher levels of CD73 compared to splenic NK cells in mice. In human gastrointestinal stromal tumor (GIST), both CD39 and CD73 exhibit higher expression in infiltrating NK cells compared to PBMC. A2AR engagement increases CD73 and decreases CD39 on NK cells (158). As depicted in **Figure 3**, A2AR, like its known influences on CD8 suppression, can be proactive in regulating NK functions in several ways: (i) it can suppress NK maturation, and (ii) A2AR-deficient or inhibited NK cells can enhance anti-tumor cytolytic and cytokine functions, and block CD73-mediated metastatic growth (157, 158).

#### 4.4.6 γδ T cells in adenosine signaling

In the lineup of anti-tumor immune players, γδ T cells often serve as a proxy for CD8 and NK cells, as well as other immune effector subsets known for HLA signaling defects that perturb the APC function, CD8, and NK cell modulation (159, 160). Since γδ T cells can act independently in these scenarios, mainly by triggering FasL and TRAIL-mediated killing, it is imperative to examine their roles in light of the adenosinergic pathway. Interestingly, CD103+CD39+γδ T cells are present in high numbers in B2M-defective CRC, where the classical antigen presentation machinery remains non-functional (160). Vγ9Vδ2T cells from healthy donors, in the presence of IL-21, undergo a mechanistic shift to the CD73-high regulatory phenotype. Through the augmentation of IL-10 and IL-8, CD73-high γδ T cell subset in co-culture with DC impairs T-cell proliferation, dampens cytotoxic and cytokine functions, and instigates defects in DC functionality by diminishing IL-12 production and T cell activation. Intratumoral γδ T cells in the syngeneic mice model express high CD73 and produce IL-10 (161, 162). Lower expression of CD73 and higher expression of A2AR, compared to regular T cells, give γδ T cells an edge in trapping eAdo, thereby reducing their access to T cells (161, 162) (**Figure 3**). Earlier experiments, however, emphasized γδ T-specific functions mainly in inflammatory diseases (163, 164). Several recent reports have indicated that CD73+ or CD39+ regulatory γδ T cells are adenosinergic in breast and colorectal cancers (58, 165, 166). The γδ T cells are essential arsenals of the adaptive immune response. Current approaches, such as CAR γδ T (153) and their A2AR inhibition, also warrant considerable safety and efficacy evaluation. In summary, γδ T cells display phenotypic diversity. The key surface markers, their involvement in adenosine-driven signaling events, and the balance between effector and regulatory phenotypes as well as drugs targeting these subsets are schematically presented in **Figure 3**.

### 4.5 A2AR and A2BR adenosine receptor network in myeloid cells

Myeloid-derived populations are diverse and have evoked considerable interest due to their regulatory roles in immunotherapies. Different populations of myeloid cells, including MDSCs and TAMs, are prominent expressors of A2AR and A2BR. A2AR and A2BR are co-expressed in macrophages. Under adenosine high condition, they outcompete TLR4-mediated TNF-α production and direct M1 to M2 shift of TAM by augmenting IL-10 production (167, 168). A2AR and CD73 co-blockade reduces M2-polarized macrophages (M2-TAM). When combined with radiation, it can reduce Treg cells in the tumor, which otherwise provide a continuous supply of eAdo and elicit suppressive effects on IL-12 production, cytotoxic cytokine production, and T cell proliferation (31, 139) (**Figure 3**). Conventional DCs (cDCs) become dysfunctional when exposed to a suppressive TME (169–171). The roles of A2AR and A2BR, and the adenosinergic network, in this dysregulation in cancers have been evaluated in preclinical settings. In this direction, an engineered mice model lacking myeloid-specific A2AR provided critical mechanistic insights. The upregulation of MHC-II and IL-2, along with a reduction of IL-10 in conventional DC and MDSC in this model, involves A2AR-deleted myeloid cells. They reinvigorate CD44+CD8 memory T cells and NK-cell-mediated IFN-γ production, effector differentiation, and lung-specific immune infiltration (172). Priming of A2BR-expressing DC with an A2BR selective agonist diminishes IL-12 production, acquires a tolerogenic state, and exhibits tumor-promoting roles in mice. A2BR gene knockout in mice reverses these properties (123). Coherently, the activation of A2BR in DC can determine their skewed differentiation into a tolerogenic (CD86 low) and proangiogenic state, as well as defective allo-stimulation under the influence of a heightened eAdo level. These, in tandem, evoke an anti-inflammatory response through VEGF, IL-10, TGF-β, and augment pro-tumorigenic chemokines such as CXCL1 and CXCL5 (103, 124) (**Figure 3**). Similarly, CD73-inducing exosomes can present adenosinergic signals to DC, inhibiting ATP-dependent TNF-α production (173). Further research gauging the diverse impacts and emerging opportunities to target adenosine receptor subtypes in DC can be insightful in developing tailor-made dual A2AR and A2BR antagonists for Ado-rich tumors.

### 4.6 Key adenosine axis and coinhibitory receptors in terminal exhaustion

**Figure 4:**
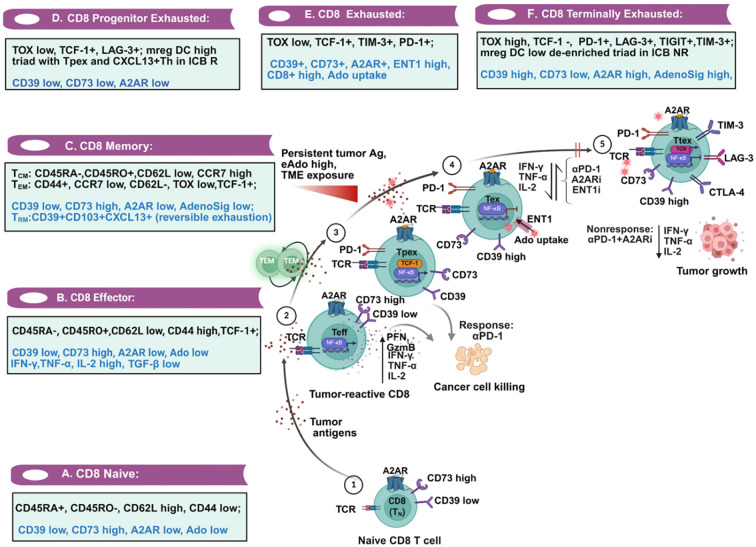
Divergent adenosine signaling network cooperates to define the steps involved in T cell exhaustion. The journey of T cells from naïve (Tn) to terminal exhaustion (Ttex) finds its link to adenosinergic and A2AR signaling, as well as its upstream regulatory mechanisms and downstream cytokine and cytotoxic functions. Key steps **(A–F)** are illustrated. It starts with an encounter of Tn with tumor antigens **(A)** and its differentiation to effector T cells (Teff) **(B)**, branching into T_CM_ and T_EM_ memory cells **(C)** or progenitor exhausted (Tpex) cells **(D)**, exhausted (Tex) cells **(E)** and final differentiation into terminally exhausted T (Ttex) cells **(F)**. The major delineators (e.g., PD-1, TIM-3, LAG-3), transcriptional regulators (e.g., TCF-1 for Tpex and TOX for Ttex), and functional mediators are presented in the corresponding box. The divergent expression patterns of CD39 and CD73, in tandem with A2AR, reveal their coordination at each step (marked in blue in each box). While Teff is usually characterized by A2AR-low, CD39-high, and CD73-low CD8+ T cells, Ttex expresses high A2AR and CD39 and is nonresponsive to therapies. The triad involving mreg DC-Teff-like CD8, and Tpex, when de-enriched, promotes nonresponse to treatment. A variable expression pattern of CD73 has been reported in different studies, primarily in Tex-like scenarios. Tn, Naïve T cell; T_EM_, Effector Memory T cells; T_CM_, Central Memory T cells; T_RM_, Tissue-resident Memory T cells; TOX, Thymocyte selection-associated HMG box; TCF-1, T Cell Factor 1; LAG-3, Lymphocyte-Activation Gene 3; TIM-3, T cell Immunoglobulin and Mucin domain 3; TIGIT, T cell Immunoreceptor with Ig and ITIM domains; mregDC, mature DC enriched in immunoregulatory molecules. Image created using BioRender.com.

ICB therapy, in essence, reinvigorates T cells at the exhaustion phase after their initial encounter with antigens. In this multi-step process, antigen-naïve CD8+ (Tn) cells undergo differentiation into either effector (Teff) or divert into effector memory (Tem) phenotypes, or evolve into terminally exhausted (Ttex) phenotypes, depending on the magnitude of antigen exposure (174). Beltra et al. described four distinct subsets of CD8 exhaustion (175). Th0, after the antigen-reactive phase, progress into central/effector memory (T_CM_, T_EM_) phenotypes. Alternatively, after initial differentiation into progenitor exhaustion (Tpex) subsets, T-ex cells are finally destined to terminal exhaustion (Ttex). Maintaining stem cell-like memory T cells (T_SCM_) is crucial for sustaining persistent memory responses and proliferation (176, 177). On the contrary, Ttex cells lose their tumor-killing efficiency dramatically. Several recent reviews elegantly presented this distinct trajectory (174, 178, 179). Distinct transcriptional factors (e.g., TCF-1/TOX), surface markers, signature cytokines, and regulatory metabolites define the exhaustion properties and tolerance of T cells (**Figures 4A–F**). Here, we discuss the complexity of the CD39-CD73-A2AR axis and its influences on effector/memory and other T cell subsets. Progress in research can improve existing as well as new-generation immune-modulating therapies.

#### 4.6.1 CD39 and A2AR in T-cell memory dysfunction

CD39 co-expressing tissue-resident memory (TRM) marker CD103 and chemokine CXCL13 in CTL represent a reversible exhaustion stage. Their density in tumor nests improves recurrence-free survival (RFS) (149). Knockdown of the *ADORA2A* in CD45RO+ memory T cells restored their chemotaxis potential in head and neck cancers (180). Paradoxically, A2AR-proficient intra-tumoral CD8 T cells can protect IL-7Rα signaling and memory phenotype. Similarly, A2AR, by downregulating FasL, helps CD4 cell survival. In brief, these strategies avert activation-induced cell death (AICD). Optimized dosing of A2AR antagonists can potentiate their safety and the durability of response (181, 182).

#### 4.6.2 CD39 in the context of adenosinergic T cell exhaustion

CD39 has a critical role in exhaustion. The absence of CD39 increases stem-like T-cells in responders to adoptively transferred T-cells (183). CD8 cells co-expressing CD39 represent tumor-reactive properties and predict clinical benefit from ICB therapy in lung cancers (184). Canale FP et al. demonstrated that elevated expression of CD39 on CD8 cells in growing tumors provokes T cell exhaustion marked by a decrease in cytokines and an increase in TIM-3 and LAG-3, and TIGIT. Notably, CD39 moderate CD8 cells also exhibit a heterogeneous distribution of naïve and central memory (185). In clinically aggressive ovarian cancers, CD39 co-expression with PD-1 and TIGIT in CD8 cells reduced the expression of T Cell Factor 1 (TCF-1). Reciprocally, thymocyte selection-associated HMG box (TOX) was frequently present in this population. A nanobody construct against CD39 restored the cytotoxic function of activated CD8+ T cells (186). CD39hi-TOXhi-PD-1hi-CD8+ T cells exhibit terminal exhaustion properties, resulting in ICB nonresponse. The progenitor-exhausted PD-1+TCF-1+ cells during ICB therapy coordinate with the CXCL13+Th subset. Maturation regulatory DCs (mregDCs) form a robust triad with Tpex and CXCL13+Th in responders, which are de-enriched in Ttex cells after treatment failure (187).

#### 4.6.3 CD73 and terminal exhaustion

CD73 has varied contributions to terminal exhaustion depending on the distribution and proximity to CD39 (185, 188). Naïve CD8+ T (Tn) cells mostly exhibit a higher level of CD73, which declines following antigenic stimulation. Elevated CD39 under this condition leads to terminal exhaustion (186). In head and neck cancers, however, Deng et al. observed a reversal of this exhaustion following CD73 blockade (189). On the other hand, in relapsed AML, established features of exhaustion were detected in CD8+ T cells, along with a decrease of CD127 and TCF-1, and an increase of TOX (190). A lower frequency of CD73-expressing CD8 cells in the blood improves the survival of melanoma patients (191). A high percentage of CD39+ exhausted CAR-T also co-expresses CD73 (71). These and other data indicate that the mere co-expression of CD39 and CD73 doesn’t dictate T cell exhaustion. Hypoxia, cytokines, lactate, co-inhibitory receptors, and non-canonical contexts of ADOR also actively participate in this event (192–194) (**Figure 4**). CD39+CD73-CD4 Treg cells can interfere with CD39-CD73+Teff function by cooperatively producing excessive eAdo from eATP (195).

#### 4.6.4 A2AR in the terminal exhaustion of T cells

The role of A2AR in terminal exhaustion is of prime interest in designing optimal intervention strategies. A2AR agonist dampens the polyfunctional CD8+T cell response and cytokines. The Gαs–PKA-cAMP cascade and subsequent induction of pCREB guide the terminal exhaustion of CD8 (196). A2AR in PBMC and TILs impairs the central memory and cytotoxic functions of tumor-expanded effectors T cells (111). In clinically aggressive BC, a higher level of A2AR expression on TILs causes disease progression and T cell exhaustion (197). Glioma expresses high CD39, CD73, and A2AR that converse with the PD-1 pathway. A2AR inhibition alone could not reverse T cell exhaustion. PD-1, LAG-3, CD38, and CD160 are also present in high numbers in their CD8 cells. The poor outcomes of A2AR inhibition in glioma plausibly emanate from an interplay of TGF-β and IL-10 (198). Like LAG-3, TIM-3 converses with A2AR, therefore impairing the CTL engagement with target antigens and killing of tumors. Combined blockade of A2AR with TIM-3 can reverse these effects and enhance tumor killing by increasing the TILs infiltration (199). Additionally, LAG3 gene editing in CAR-T showed a stable phenotype and profound tumor-killing effects (200). Opdualag (relatlimab+ nivolumab) has recently been approved for advanced or metastatic melanoma (201). A2AR-selective antagonist CPI-444 also downregulates PD-1 and LAG-3 on Teff and preserves rechallenged memory response (202). Therefore, it is crucial to identify the context-specific exhaustion phenotypes for ascertaining a biomarker-guided combination option. Clinical data also revealed that the LAG-3 inhibitor improves ORR in both treatment naïve and previously anti-PD-1 treated nasopharyngeal cancers with elevated LAG-3 (203). Moreover, single-agent CPI-444 caused the shrinkage of refractory tumors, suggesting the potential benefits of targeting multiple checkpoints (204–206).

The adenosine signaling score revealed a significant positive correlation with CD8 and NK cell exhaustion in human cancers. A drop in this score was observed in patients in a phase 1 trial of AZD 4635 with a concomitant increase in IFN-γ and cytotoxicity (207). The co-dependency of TAM and Tex stems from the transcriptional and epigenetic program of CD8+ TILs that release chemokines, recruiting monocytes. CD8+T cells and monocyte-derived TAMs form stable synapses, which prominently contribute to CD8 exhaustion in the hypoxic area of the tumor (208). Similarly, SPP1-high TAM enrichment score and CD8+ T cell exhaustion score, or AdenoSig score and T cell exhaustion score, showed a positive correlation in aggressive mCRPC (209). Ado uptake by activated CD8 cells through ENT1 can influence terminally differentiated T cells and memory T cell responses by impairing mitochondrial respiration and pyrimidine biosynthesis. A potent ENT1 antagonist, EOS301984, reverses these functions and synergizes with an A2AR antagonist and a PD-1 inhibitor in preventing growth upon rechallenge (39). **Figures 4A–F** presents an overview of the key factors that underscore the plasticity of effector, memory, and exhaustion phenotypes. These studies appreciate that the heterogeneity of A2AR and other co-inhibitors underscores an interplay of these regulators in defining terminal exhaustion.

## 5 Cancer-specific tumor immune microenvironment and A2AR signaling

Spatiotemporal heterogeneity of TIME is a key determinant of heterogeneous response patterns of immunotherapy (210–212). This heterogeneity was observed in patient-derived tumor models (213). Tumor cell inherent factors, TILs, along with spatial immune contexture, contribute to the variable response trajectories to immunotherapy (214–216). There is growing enthusiasm in examining TIME as a dynamic and actionable milieu. TME- or TIME-guided ex vivo models preserve tumor heterogeneity and complement the roles of biomarkers (217–219). A mechanistically divergent and collaborative adenosine signaling network intertwines with this heterogeneity (220, 221). Therefore, narrowing the focus to local TIME and its adenosine environment offers a logical approach to characterize unique cancer subtypes. The TIME of multiple cancer types that exhibit unique molecular phenotypes and genomic signatures is crucial for therapy response. This diversity provides a selective advantage, enabling the personalization of the modalities that were not considered before. We discuss the nuances and unique contexts of three major cancer types and present their A2AR perspectives.

### 5.1 TIME in hepatocellular carcinoma

**Figure 5:**
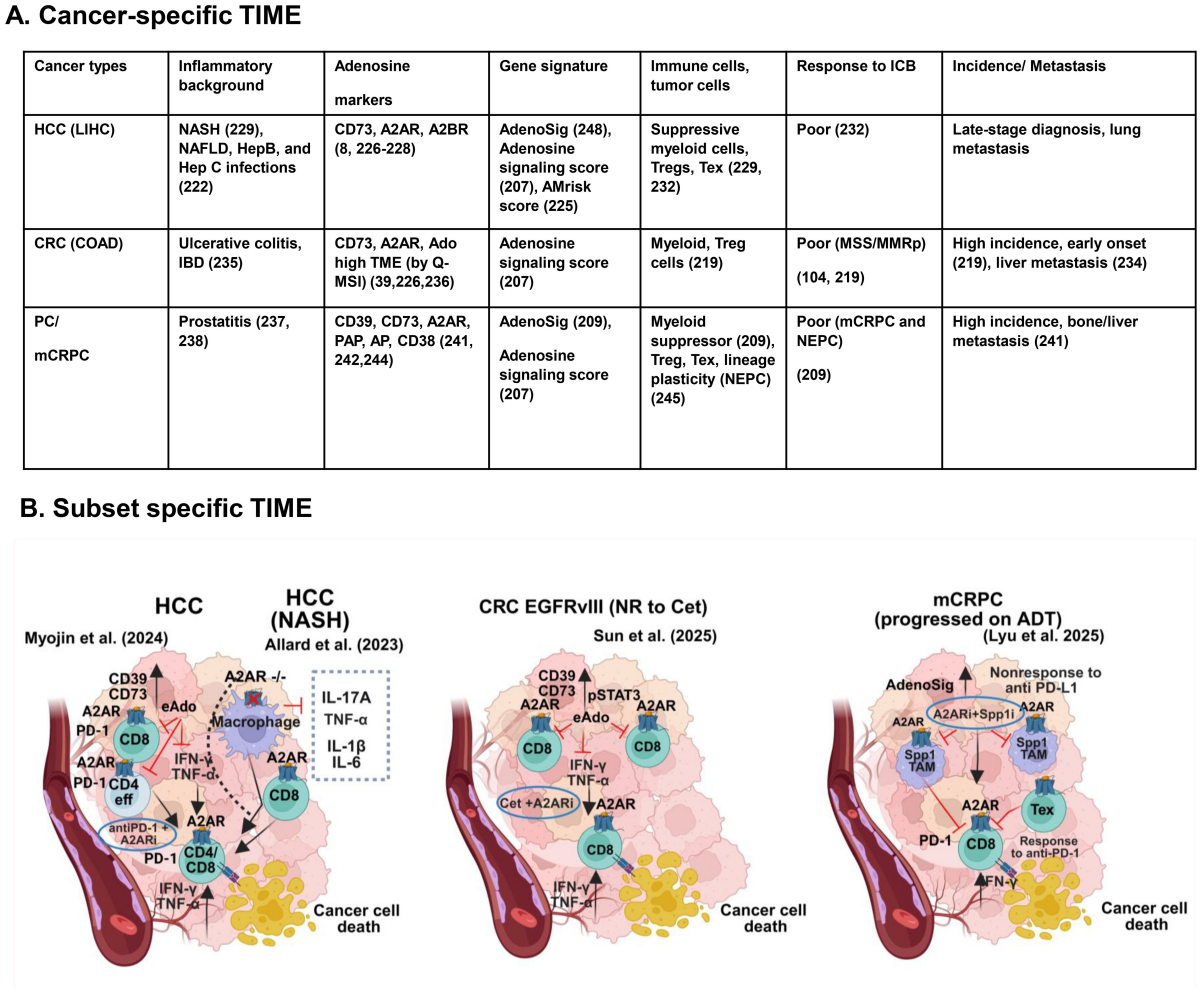
Cancer-type-specific TIME contexts and subtype-specific novel intervention. **(A)** Disease-specific nuances in HCC, mCRPC, and CRC highlight context-dependent roles of adenosine signaling. Key references are shown in brackets. **(B)** TIME context guides subtype-specific and biomarker-guided novel intervention. A2AR signaling drives a unique niche in TIME as represented by HCC, including NASH-HCC, CRC, and mCRPC. Key cell types and their immune interaction networks are depicted along with personalized therapy options. Blue circle indicates TIME-guided therapy. A black arrow with a blunt end indicates the downstream inhibitory effects of A2AR signaling. The red arrow with a blunt end indicates interventions by a particular therapy. LIHC, Liver Hepatocellular Carcinoma; HCC, Hepatocellular Carcinoma; NASH, non-alcoholic steatohepatitis; NAFLD, Nonalcoholic Fatty Liver Disease; IBD, Inflammatory Bowel Diseases; PC, Prostate Cancer, mCRPC, Metastatic Castration-Resistant Prostate Cancer; MSI, Mass Spectrometry Imaging; AMrisk, Adenosine Metabolism-related risk, NEPC, Neuroendocrine prostate cancer; COAD, Colorectal Adenocarcinoma, CRC, Colorectal Cancer; MSS, Microsatellite Stability; MMRp, Mismatch Repair-proficient; EGFRvIII, Epidermal Growth Factor Receptor variant III; MDSC, Myeloid-derived Suppressor Cells, M2-TAM, Type 2-Tumor-Associated Macrophages; Cet, Cetuximab; IRI, Irinotecan; POM, Polyoxometalates; Spp1, Secreted Phosphoprotein 1; ADT, Androgen Deprivation Therapy; Tex, T cell exhaustion. Image adopted from ‘Clocking Cancer Immunotherapy Responses, Catherine L. Wang,’ using BioRender.com.

HCC pathogenesis is linked to nonalcoholic fatty liver disease (NAFLD), nonalcoholic steatohepatitis (NASH), hepatitis B infection, and liver cirrhosis. The advanced stage and multifocal distribution make its therapeutic management challenging (222). TIME components of HCC present a dynamic context (223). TIME-based therapies hint at the vulnerability of A2AR signaling. The tumor, stroma, and immune cells of TIME in HCC augment local adenosine signaling (224). A six-gene adenosine metabolism-related risk score (AMrisk), complemented by immune checkpoints and infiltration of aberrantly activated immune cells, stratified the risk of poor survival (225). While genomic heterogeneity is a key barrier, CD73, A2AR, and A2BR showed higher expression in HCC and regulate its invasiveness (8, 226–228) (**Figure 5A**). A2AR deficiency in mice promotes inflammation-driven NASH and its progression to HCC. In this context, HCC patients with low A2AR expression have a poor prognosis. Additionally, patients with a NASH background respond poorly to A2AR-targeted treatments compared to those without NASH. A temporal switch in A2AR protects tumor hosts from disease progression by suppressing the macrophage-mediated release of IL-17A, TNF-α, IL-1β, and IL-6 (229) (**Figure 5B**). Higher expression of *ADORA2A* was correlated with non-responders to PD-1 inhibitors. This data needs further validation in a larger population (230). Earlier study showed hypoxia-CD39 impaired MDSC differentiation to DC (231). Similarly, higher expression of A2AR, CD39, and CD73 was observed in the peripheral compartment of PD-1-positive CD4+ and CD8+ T cells. A2AR and CD39 exhibit higher expression in proliferating CD4 cells, regardless of PD-1 status. However, this pattern persists only in PD-1-negative proliferating CD8 cells in the HCC after anti-CTLA-4 therapy. Liver-derived A2AR+CD39+CD73+CD4 and A2AR+CD39+CD8 T cells are abundant in the HCC of mice. Anti-PD-1 treatment further elevates A2AR and CD39 levels in CD8 cells. The combined blockade of A2AR and PD-1 increases the proportion of CD4+ and CD8+ T cells and elicits a synergistic response (232) (**Figure 5B**).

### 5.2 TIME in mismatch repaitr-proficient colorectal cancer with EGFRvIII

Pathogenesis of CRC is connected to colitis-driven inflammatory bowel diseases (IBD), demonstrating poor prognosis and a higher risk of liver metastasis (233–235). Defects in the adenosine signaling can support colorectal tumorigenesis. Higher expression of CD73 and A2AR genes and unfavourable prognosis instigate the need to dissect their roles as therapeutic targets (8, 226, 236). Using qMSI, Sanders et al. demonstrated a heterogeneous distribution of adenosine across six cancer types, with CRC and endometrial cancer showing the highest intensity (39). A2AR deletion synergies with anti-PD-1 agent in executing an anti-metastatic response (156, 157). PEGylated-ADA, by reducing adenosine levels, boosts TILs and restricts tumor growth (67) (**Figure 5A**). MMRp/MSS subtypes of CRC are notoriously immune-cold and associated with poor prognosis. There’s an unmet need to understand molecular biomarkers, therapy resistance, and new combination therapies for this patient population (219). Data from a multi-center cohort study revealed that 10% of CRC patients have EGFRvIII (an EGFR variant known for its prevalence in GBM). Because of the loss of the extracellular ligand-binding domain, tumors with this variant don’t respond to cetuximab. They express high pSTAT3, leading to upregulated CD39, CD73, and eAdo levels, diminished IFN-γ, TNF-α, and TILs. The high adenosine background in these CRCs forms the basis for testing cetuximab and an A2AR antagonist. A2AR inhibitor ZM241385 restores CD8 function, allowing them to produce IFN-γ and TNF-α. This novel combination not only reverses cetuximab resistance and remodels the TME but also enhances anti-PD-1 effects. A four-drug combination of cetuximab, irinotecan (IRI), POM-1(a CD39 inhibitor), and anti-PD1 resulted in higher efficacy in mice MC38 tumors (104) (**Figure 5B**). We recently reviewed spatiotemporally unique TIME and systemic immune landscapes of MMRp and MMRd-specific CRC (219).

### 5.3 TIME in metastatic castration resistant prostate cancer

Prostate cancers (PC) have an inflammatory connection (237, 238). They not only express higher levels of adenosine gene signatures and CD39, CD73, but also utilize non-canonical enzymes like CD38-CD203a, PAP, and AP (239–243). The mCRPC is a lethal state with a high risk of bone metastasis. Higher expression of CD39 and CD73 is associated with bone metastasis (241, 244). It often undergoes lineage change to develop an aggressive androgen receptor (AR) independent, PSA/PSMA loss phenotype (245). Due to low PD-L1 expression, mCRPC showed a poor response to PD-1 blockade, but showed a modest response to A2AR (246–248). TIME is a mechanistic barrier that involves active interference by suppressive immune cells (**Figure 5A**). Bridging this gap, single-cell RNA profiling of biopsied tumors identified a new myeloid subset in mCRPC. A novel TAM subset expressing Spp1 transcripts was abundant in these tumors. Spp-high TAMs are functionally different from conventional TAMs. They orchestrate PD-1 resistance and are nonresponsive to anti-CSF-1R treatment. Spp1-high TAMs revealed a mechanistic link between this phenotype and AdenoSig high and high T-cell exhaustion scores. Combining an A2AR antagonist with PD-1-specific blockade successfully eliminated Spp1-high TAMs and restored responsiveness to anti-PD-1 therapy (209) (**Figure 5B**).

## 6 ENT1, MTAP loss, and other alternative vulnerabilities

Besides the foundational A2AR and A2BR agonists generated by the CD39-CD73 axis, multiple non-canonical and nonredundant nodes regulate adenosine generation and signaling pathways. Further understanding of these modules will provide coherent insights for developing next-generation therapies.

### 6.1 Intervention of ENT1 in adenosine transport

Ado uptake by activated CD8 cells through ENT1 can act as a hurdle for terminally differentiated T cells and memory T cell responses. It impairs mitochondrial respiration and *de novo* pyrimidine biosynthesis, a key modulator of T cell proliferation. A potent ENT1 antagonist, EOS301984, reverses these functions. Concurrent blocking of A2AR by its antagonist (inupadenant) and limiting intracellular eAdo concentration by EOS301984 synergizes with a PD-1 inhibitor, preventing tumor growth upon rechallenge in a humanized TNBC mice model (39).

### 6.2 Targeting MTAP loss and MTA-induced immune dysregulation

Growing interest in understanding ‘difficult to drug’ molecular targets sheds light on Methylthioadenosine Phosphorylase (MTAP) loss (249). MTAP gene and protein loss is common in 10-15% of all cancers and causes a poor prognosis (250–253). Although varied amoung cancers, more than 80% of MTAP-loss cancers also display codeletion of *CDKN2A*. Its independent prognostic value, however, has gained attention only recently (249–251). MTAP loss leads to the accumulation of methylthioadenosine (MTA). MTA acts at the intracellular level by inhibiting the MAT2A/PRMT5/RIOK1 axis, thereby weaponizing this pathway for PRMT5 vulnerability in cancer cells, and synergizing with pemetrexed (254–256). MTAP, in its intact form, converts MTA to methylthioribose-1-phosphate (MTR-1P) and adenine. MTA has a structural resemblance to Ado. MTAP loss variant releases MTA into the extracellular space. Its subsequent binding to A2AR and A2BR drives M2 macrophage polarization (253, 257), as well as defects in T cell and NK cytolytic and cytokine signaling (258–260). It also impairs DC maturation, suppresses IL-12 production, and inhibits T cell activation (261). MTAP/9p21 loss is tightly linked with low B cell, CD8, and CTL density, as well as their diminished trafficking and activation, and resistance to ICB therapy (262). Additionally, tumor-derived MTA, through arginine methylation of STAT1, impairs the type 1 IFN response (263). Consistent with these observations, a recent report confirmed that while A2AR and A2BR blockade by their antagonists shows moderate restoration of MTA-induced inhibition of CD4 and CD8 proliferation in the MTA loss model, direct degradation of MTA by PEGylated MTAP can rescue CD8-dependent anti-tumor response more potently and sensitizes tumor cells to ICB therapy (259).

### 6.3 Actionability of ADA and ADK

In 1985, human genetic analysis discovered that ADA mutations are related to immunodeficiency in newborns (264). Loss-of-function mutations in the ADA gene led to lymphopenia and impaired T-cell proliferation (265). The modulatory roles of ADK and ADA in adenosine metabolism have been examined in different cancer types. Acidic TME is a barrier to efficient drug action. The ADA2 isoform at acidic pH converts Ado to INO (66). PEGylated ADA2 depletes Ado, inhibiting tumor growth (67). ADK inhibitor ABT-702 restores T cell proliferation in Ado-complemented environment by disrupting the Ado and AMP partnership that mediates suppressive effects on pyrimidine biosynthesis (39).

### 6.4 CD38-ENPP1-axis: non-canonical adenosinergic checkpoints

Non-canonical adenosinergic axis offers several potential mechanistic targets (8, 36, 37, 266). CD38 is a theranostic target in both haematological and solid malignancies (267). Elevated expression of CD38 is an actionable target in prostate cancer (268). Daratumumab is an approved drug for multiple myeloma, which depletes CD38+ Tregs while expanding T-cells; CD38 predicts ICB response (269–271). The eventual resistance to first-generation anti-CD38 agents has prompted the development of new therapeutics for other similar targets. ENPP1/CD203a shows a heterogeneous expression in immune cells, with predominant expression in NK cells, cDC subtypes, and mucosal-associated invariant T cells (MAIT) (272). Circulating tumor cells enriched with ENPP1 are capable of self-seeding and are associated with increased risk of relapse in breast cancer. RNA silencing of ENPP1 prolonged the relapse-free survival (273). The cell-associated and EV-bound ENPP1 reduced STING ligand cGMP, thereby diminishing type 1 interferon response (274, 275). ENPP1 inhibitor, STF-1623, is a new agent that achieves a property of ultralong tumor residence coupled with rapid systemic clearance. Through tumor-specific STING signaling, it blocks tumor progression in mice (276). An AI-driven design of the ENTPP1-selective, orally bioavailable inhibitor ISM5939 modulated STING and demonstrated synergy with chemo agents and PD-1/PD-L1 inhibitors. While the study found no loss of viable TILs, and there was no off-target inflammation, it showed an exuberance of bystander antigen-presenting cells. Interestingly, ISM5939-mediated ENPP1 inhibition has diminished adenosine production with potential impact on TILs (277).

## 7 Clinical trial landscape of the A2AR and A2BR pathway

Immunomodulatory drugs, acting against the adenosinergic axis and the A2AR/A2BR pathway, have been evaluated for safety and efficacy in advanced-stage malignancies (8). Inhibitors of the CD39/CD73, as well as their combinations with either immunotherapy, chemotherapy, or bifunctional traps with TGF-β, are under clinical evaluation (226). Other agents targeting A2AR, A2BR (NCT05234307), or dual A2AR-A2BR (NCT05024097) are in clinical trials, primarily in combinatorial settings, for several indications (239) (**Table 1**). Preliminary safety and efficacy data from some ongoing and completed trials are available (**Tables 1** and **2**). Safety profiles like treatment-related adverse events below grade 3 (TRAE<grade 3) in most cases are manageable. However, a wide range of objective response rates (ORR) or disease control rates (DCR) has emerged due to the divergence of drugs, contexts, and indications (**Tables 1** and **2**). A first-in-human multicenter trial of a potent A2AR and A2BR dual antagonist (ADPORT-601, NCT04969315) measured serum mRNA levels of A2AR and A2BR. The trial detected A2AR enrichment in patients with bone metastasis compared to patients with localized diseases. More importantly, the same trial measured image-guided levels of key Ado precursors and metabolites in bones. Further translational analysis using multi-omics platforms is in progress. A complete analysis will shed light on the elusive biomarker aspects (29). A phase I trial evaluated AZD4635 (A2AR antagonist) as monotherapy or in combination with durvalumab in naïve or previously treated NSCLC, CRC, and mCRPC. AZD 4635 monotherapy showed anti-tumor responses in 2/39 prostate cancer patients. Combination therapy showed the same effects in 6/37 (one CR) patients. It also led to a drop in serum PSA level. Peripheral blood adenosine signature at baseline predicted PFS (21 weeks in high vs. 8.7 weeks in low signature) (248). An aggravated eAdo level following RT dampens the RT-induced IFN1-mediated anti-tumor responses. PANTHER RC trial hypothesized that administering A2A and A2B receptor dual antagonist AB928 is a safe and effective strategy to boost RT-induced anti-tumor T cell functions. The combination of PD-1 inhibitor (AB122) with AB928 plus CT after short induction by RT in locally advanced rectal cancer showed improved pCR, cCR, and cPR (278). In the recently presented ARC-9 study (NCT04660812), a combination of AB928 with anti-PD-1 (zimberelimab), FOLFOX, and bevacizumab resulted in better PFS and OS compared to the regorafenib control arm in third-line, chemo-resistant mCRC (with baseline CD73 expression>1% in tumor cells). The primary analysis also showed that this regimen significantly reduced the expression levels of the adenosine-regulated NR4A 1,2,3, accompanied by an increase in T cell inflammation (288).

**Table 1:** Ongoing immuno-oncology clinical trials for drug candidates targeting the adenosine pathway, including safety and efficacy updates.

Phase, size (N), status	Cancer type	Agents	Targets	Design and endpoints measure	Safety (TRAEs)	Efficacy		Tria ID, reference
						ORR/DCR (%)	PFS/OS	
Phase Ib/II, (N = 24), Recruiting	Advanced RCC	Ipilimumab, Nivolumab, and Ciforadenant	PD-1, CTLA-4, A2AR	Interventional, Open Label. Primary: Tolerability, depth of response. Secondary: ORR, DOR, PFS, PD, irAE.	Phase 1b: ciforadenant,100 mg BID orally, nivolumab 3 mg/kg, and ipilimumab 1 mg/kg q3 weeks. Phase 2 dose-expansion. TRAE (>Grade 3): 34% (of n=50 evaluable cases). Common TRAE in all grades: fatigue, rash, AST and ALT increase, hyponatremia. One patient died of ICI overlap syndrome. Overall: acceptable safety from triplet therapy.	Follow-up: 9.4 mo. >50% tumor shrinkage: 32%. ORR: 46%. PD as best response:20%. *Signatures: Fong et al. (2000): no response improvement. B: IO cluster: ORR: 100% (n=4) vs 30% (n=21) in Angio cluster, (p=0.032 and correlated with PFS.	mPFS: 8.5 mo: no further improved efficacy in triplet therapy.	NCT05501054. ESMO Congress 2025, Abstract 2596MO.
Phase I (N = 30), Recruiting	Recurrent or mNSCLC	PBF-1129, Nivolumab	A2BR, PD-1	Open-label, Primary: AE Secondary: ORR, DCR, OS, PFS Others: level of MDSC, correlative biomarkers.	N/A	N/A	N/A	NCT05234307
Phase II, (N = 186), Completed	Locally advanced NSCLC	Inupadenant, Carboplatin, Pemetrexed	A2AR	Interventional. Randomized. Parallel Assignment. Primary: Dose, PFS, AE. Secondary: Cmax, Tmax, AUCinf, OS, ORR, DCR, T1/2, PRO.	No death, TRAEs: In the line of doublet chemotherapy.	ORR: 63.9 (all cohorts), 53.% (Inupadenant 40mg), and 73.3% (Inupadenant 80mg).	PFS (6 Mo): 5.6 mo (Inupadenant (40 mg). 7.7 mo for 80 mg dose.	NCT03873883 (www.esmoiotech.org/article/S2590-0188(24)00046/7/pdf)
Phase I-II, (N = 43), Recruiting	Rectal Cancer	Etrumadenant, RT, FOLFOX, Zimberelimab	A2AR and A2BR, PD-1	Open Label Primary: CPR, CCP. Secondary: CTAEA, PFS, OS.	TRAE (Grade 3+): 1/6 in Part I (RT+SC).	CR: 20%, in Part I, CR: 82% (4 pCRs, 5 cCRs, 2 pPRs) in Part II.	N/A	NCT05024097 (PANTHER), (278).
Phase I/Ib, (N = 180), Active	Advanced/Relapsed RCC, other cancers	DFF332 (HIF2α inhibitor), Everolimus, Spartalizumab, Taminadenant	HIF-2alpha mTOR, PD-1, A2AR	Open-label, Parallel Assignment, Non-Randomized. Primary: AE, dose intensity. Secondary: ORR, BOR, PFS, DOR, DCR Cmax, AUC.	For DFF332 (mono), TRAE (Grade 4): None, Grade 3: N = 3/40. Overall TRAE: 62.5%. Anemia in 32.5%, fatigue in 37.5. Grade 3 TRAEs: weight gain and hypertension.	For DFF332 (mono), BOR: 52.5%. PR: 5%. SD: 47.5%.	N/A	NCT04895748, Pal SK et al (286).
PhaseI (N = 84), Active, not recruiting	Advanced solid tumors	EOS-984, Anti-PD-1 monoclonal antibody	ENT1, PD-1	Interventional, Open-label, Sequential. Primary: Tolerability, AE. Secondary: AUC, Cmax, RP2D, OR (RECIST v1.1).	N/A	N/A	N/A	NCT06547957
Phase I, II (N = 90), Recruiting	RCC, CRPC, NSCLC, HNSCC, CRC, EC, OC	A2AR and A2BR single and dual blockade		Open-label, Non-Randomized, Sequential. Primary: DLTs, MTD, safety (TRAEs). Secondary: ORR, DOR, PFS, Cmax, AUC, T1/2.	TRAEs (G1-2): nausea (29%) and vomiting (14%). SAE/DLTs: None.	N/A	N/A	NCT04969315 (ADPORT-601) (287).
Phase I, II (N = 217), Completed	mCRC	Etrumadenant, modified FOLFOX6,zimberelimab	BRAF mutant negative	Randomized, Parallel Assignment Primary: PFS, ORR Secondary: ORR, DOR, DCR, OS, Cmax	TRAEs (Grade ≥3): 82.4% (EZFB) vs 48.6 (Rego). Safety profile: consistent with the known FOLFOX/bev profile. EZFB or Rego: No death.	17.3% in EZFB (combo) vs 2.7% in Rego.	mPFS(mo):6.2 (EZFB) vs 2.1(Rego). mOS (mo): 19.72 (EZFB) vs 9/7 (Rego).	NCT04660812 (ARC-9) (288).

NSCLC, Non-Small Cell Lung Cancer; mNSCLC, metastatic Non-Small Cell Lung Cancer; RCC, Renal Cell Carcinoma; EC, Endometrial Cancer; OC, Ovarian Cancer, ENT1, Equilibrated Nucleoside Transporter 1; pCR, pathological Complete Response; CCR, clinical Complete Response; PR, Partial Response; PD, Progressive Diseases; AE, Adverse effects; ORR, Objective Response Rate or Overall Response Rate; OS, Overall Survival; PFS, Progression-Free Survival; DCR, Disease Control Rate; DOR, Duration of Response; RECIST, Response Evaluation Criteria in Solid Tumors; AE, Adverse Event; irAE, immune-related Adverse Event; PRO, Patient Reported Outcome; TRAE, Treatment-related adverse events, DLT, Dose Limiting Toxicity; SAE, Serious Adverse Event; MTD, Maximum Tolerated Dose; Cmax, Plasma Maximum Concentration; T max = the time taken to reach the maximum concentration; BOR, Best Overall Response; RP2D, recommended phase 2 dose; T1/2, half-life; AUC, area under curve; AUCinf, area under curve-infinity; EZFB: Etrumadenant (E)+zimberelimab (Z)+FOLFOX/bevacizumab (bev). *RNA signatures: Adenosine Signature (Fong et al., 2000) (204), IO cluster (Mmotion 151/OPTIC IO, Motzer et al., 2022) (285). For further details of all the trials listed here and their updated recruitment status, please refer to ClinicalTrials.gov.

**Table 2:** Trial design, safety, and efficacy outcomes from adenosine receptor antagonist trials completed in oncology.

Phase, enrollment (N)	Cancer types	Agents and combo partners	TRAE (%)	Response (%) (ORR, DCR, PFS, OS)	Trial ID, reference
Phase I/Ib, (N = 502)	Refractory RCC	A2AR-SMI (Ciforadenant)+ Atezolizumab (anti-PD-L1 mAb)	TRAE (Grade 3-4): 1-3% in Ciforadenant mono vs ≤3% in combo. Decreased appetite, anemia, peripheral edema (mono); Nausea, hypophosphatemia, abdominal pain (combo).	DCR (6 mo): 39% in combo vs 17% in Ciforadenant; PR:11% in combo vs 3% in mono; ORR 11% (combo) vs 3% (mono); PR:11% (combo) vs 3% (mono); SD/DCR: 39% (combo) vs 17% (mono); PFS: 5.8 mo (combo) vs 4.1 mo (mono); OS: 25 months >90% (combo) vs 69% >16 mo (mono).	NCT02655822 (MORPHEUS), Fong L et al. (204).
Phase I, (N = 92)	NSCLC	A2AR-SMI (Taminadenant) (PBF509/NIR178) +spartalizumab (anti-PD-1 mAb)	TRAE (Grade 3): 4% in taminadenant mono and 8% in combo. Mono: Alanine/aspartate aminotransferase increase and nausea; Combo: Pneumonitis (controlled with steroids), fatigue, and alanine/aspartate aminotransferase increase.	ORR (95% CI):9.5% in combo vs 8.3% in mono; DCR (95% CI):66/7% in combo vs 42.9% in mono; SD: 56% in combo vs 28% mono; CR: 4% in both arms, PR:4% in both arms, PD: 32% in combo vs 48% in mono.	NCT02403193, (289).
Phase I, (N = 313)	NSCLC, Refractory mCRPC, CRC	A2AR-SMI (AZD4635) +Durvalumab (anti-PD-L1 mAb)	TRAE: n=3: nausea, fatigue, vomiting, loss of appetite, diarrhea dizziness,	ORR: 16.2% in combo vs 6.1% in AZD4635 mono; 2CRs, 4PRs of N = 37 of mCRPC in combo.	NCT02740985, (290).
Phase I, (N = 117)	NSCLC, HNSCC, CRC, mCRPC, RCC	CPI-006 (anti-CD73)± Ciforadenant, Pembrolizumab	TRAE: CPI-006: Grade 1 infusion reactions within 30 minutes of the first infusion (controlled with NSAID).	Lymphocyte redistribution: reduction of circulating CD73pos B cells, increase in CD4:CD8 ratio and effector/memory in blood.	NCT03454451, (291).
Phase Ib/II, (N = 173)	mCRPC	AB928 (A2AR and A2BR dual antagonist) + Zimberelimab (anti-PD-1 mAb)/ Quemliclustat (anti-CD73mAb)/ Docetaxel/Sacituzumab/Govitecan	TRAE: Grade 3 in (EZD combo): 53% All grades: 88%. Lymphocyte count decrease and neutrophil count decrease (41%), hyponatremia, and alopecia (35%).	Composite response rate (EZD combo) (DCR): 43% (6/14). PSA response rate: 36% (5/14). Radiological response rate: 38% (3/8; 1 CR).	NCT04381832 (ARC6) (292).

NSCLC, Non-Small Cell Lung Cancer; RCC, Renal Cell Carcinoma; CRC, Colorectal Cancer; mCRPC, metastatic Castration resistant Prostate Cancer; TRAE, Treatment-related adverse events; NSAID, Nonsteroidal anti-inflammatory drug; CR, Complete Response; PR, Partial Response; SD, Stable Disease; PD, Progressive Disease; ORR, Objective Response Rate (CR+PR); DCR, Disease Control Rate(CR+PR+SD); OS, Overall Survival; PFS, Progression-Free Survival; PSA, Prostate Specific Antigen. For further details of all the trials listed here, please refer to ClinicalTrials.gov.

The outcomes of early and recently completed trials raised both optimism and caution. The number of registered trial, encouraging safety profiles, DCR, and ORR, indicates the value of the adenosine pathway as a promising therapeutic target. Moreover, the majority of studies enrolled advanced-stage patients, who are known to have less favourable outcomes. The efficacy data, however, did not show statistically significant deep and durable response (CR) beyond SOCs, suggesting the scope for further improvement. Despite convincingly linear PK-PD data, the benchmark response is elusive for several potential reasons: i) uncertainty over antagonists stably occupying receptors for a long time in a high adenosine environment, and ii) incomplete understanding of pathway and action mechanisms in a complex and dynamic milieu (279–281) (**Tables 1** and **2**). These limitations instigate adopting a multipronged approach to reduce Ado generation, metabolism, trafficking, and prevent their binding to receptors (39, 72, 130, 276, 277). The evolutionary divergence of PD-L1 underpins its non-linear function in the mice and human systems (282). Reliance on *in vitro* and *in vivo* models that lack the complexity of the human tumor-immune system and heterogeneous biomarker landscape further handicaps the stratification and trial designs (219, 283, 284).

## 8 Emerging biomarker landscape and predictive tools

Current trends of modest yet significant success of adenosine pathway inhibitor trials reflect the pressing need for a change in this landscape (293). A biomarker program aligned with the drug action mechanism is crucial in integrated drug development. The PD and response-prediction biomarkers are essential tools that can guide the right treatments to the right patients (8, 204, 294). One upfront challenge is the real-time monitoring of Ado levels in clinical samples due to its short (10 seconds) half-life (69). Another hurdle is the lack of optimal flow cytometry detection reagents for A2AR and A2BR. Mastelic-Gavillet B et al. showed a higher percentage of A2AR than A2BR in gated CD8 cells using both RNA-flow and flow cytometry (111). Another study reported technical challenges in quantifying A2AR in T cells due to the scarcity of a suitable flow cytometry antibody (295). LCMS, qMSI, single-cell RNA-seq, and mPlex IHC offer efficient measurement of Ado and surrogate markers in both dissociative and non-dissociative contexts (26, 31, 39). These multiplex spatial and multimodal biomarkers can efficiently address the current bottleneck of patient stratification and response prediction.

We analysed the current standings of these biomarkers in adenosine pathway inhibitors. The evolving landscape mainly focuses on a select number of assays and signatures. Blood-based pCREB assay and NR4A levels, an eight-gene adenosine gene signatures (AdenoSig), and a 14-gene adenosine signaling score, inform target engagement (PD) and response prediction (204, 207, 296). An exploratory risk stratification method (AMrisk score) has been reported for a similar purpose (225). AdenoSig showed response-predictive value, particularly in RCC, for the A2AR antagonist, but limited utility for other cancers (204), suggesting the need to integrate additional stratification tools (285). The AdenoSig and adenosine signaling score correlated across multiple cancer types and matched with the biological Ado levels (239). Patients with high adenosine signaling score showed reduced PFS and OS, decreased T cell infiltration, IFN-γ, and cytotoxicity in ICB non-responder tumors. Notably, in ICB responders, a decline in the adenosine signature is correlated with a concomitant increase in cytotoxicity and IFN-γ. A high adenosine signaling signature in tumors with high CD8+ TILs was associated with low PFS and poor OS (207).

Phospho-CREB has recently gained significant attention as a potent surrogate biomarker. At the plasma maximum concentration (Cmax), the dual A2AR and A2BR antagonist (AB928) significantly decreased the CD8-specific pCREB, with 90% inhibition observed at 1 μM or higher doses. Analysis of whole blood from AB928-treated normal donors, when treated with NECA ex vivo, showed a reversal of the agonist-induced increase in pCREB signal after 24 hours (30). A similar pattern was observed after ciforadenant treatment (204). P-CREB has been implicated in CD8 exhaustion in tumors following biomechanical stress (297). A2AR can activate NR4A1, 2, and 3, thereby attenuating the TLR4- signaling and NF-κB-induced inflammatory responses (296). NR4A2 showed upregulation in intra-tumoral Tex cells compared to naïve CD8 cells in metastatic melanoma (298). A multimodal exploratory biomarker readout of pCREB and NR4A1,2,3 will inform about: (i) the pharmacodynamic engagement of a drug with its target and downstream effects, and (ii) the status of the CD8 cell activation.

## 9 Smart design and delivery strategies of adenosine inhibitory agents

Next-generation immunotherapies face the roadblock of achieving tumor-targeted delivery and deep systemic responses due to the multi-layered barrier created by the tumor. Smashing this barrier and programming the immune cells to overcome it are unresolved needs to improve the therapy outcomes. Intra-tumoral delivery of immune-targeted agents is poised to enhance the efficacy of adoptive T cell transfer and CAR-T cells (299–301). TIME-modulating nanomaterials that combine cytotoxic and immunotherapy agents have shown encouraging results in remodeling TIME.

### 9.1 Nanoplatform-enabled drug delivery

**Figure 6:**
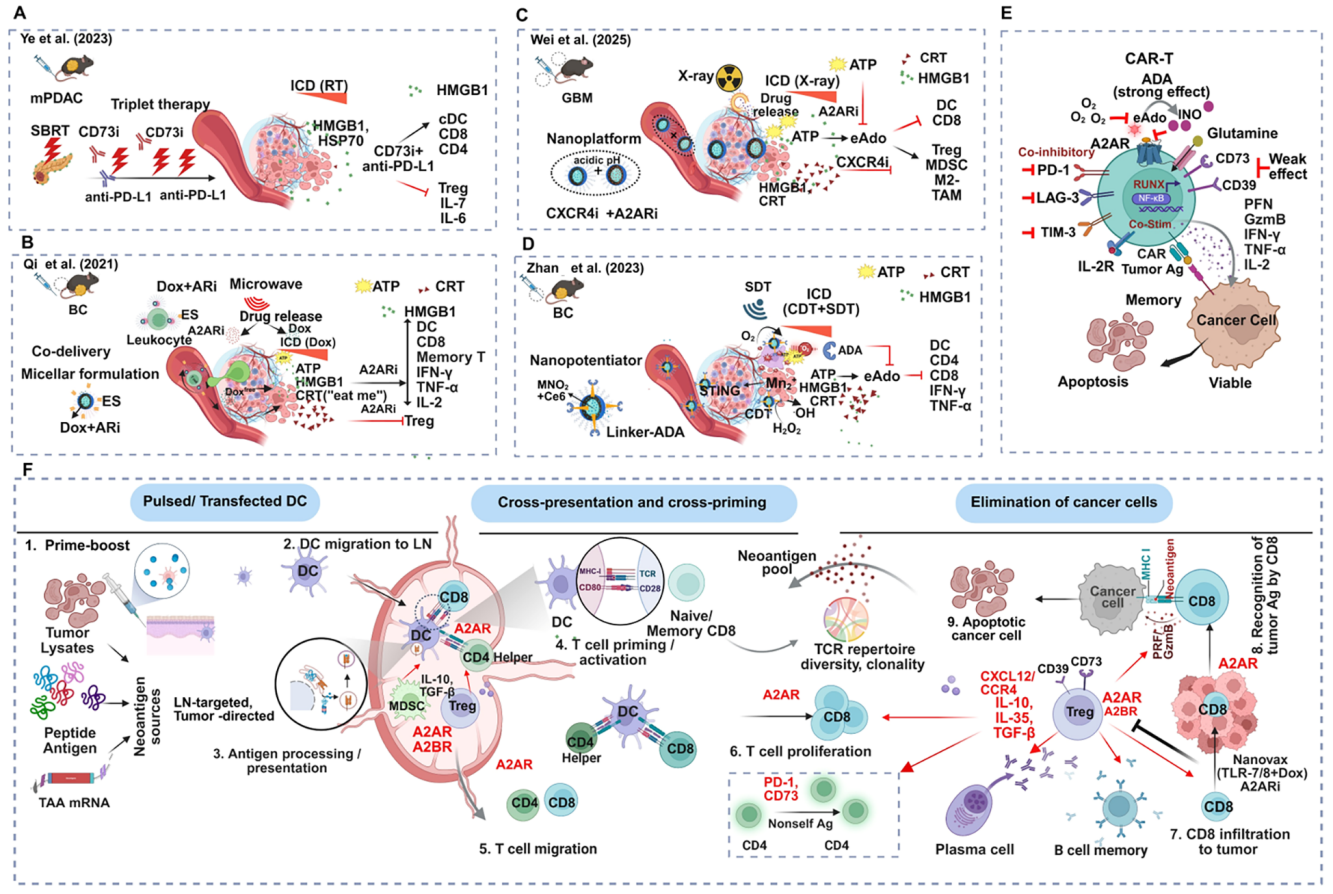
Adenosine constraints define smart delivery platforms; A2AR positioning in CAR-T and cancer vaccines. Nanoplatforms highlight the advantages of smart co-delivery of drugs targeting TIME and enhancing efficacy of anti-CD73 **(A)**, adenosine inhibitors (A2ARi) **(B, C)**, and adenosine depleting enzyme (ADA) **(D)**. They confer synergy through the induction of ICD. Tumor-directed cytotoxic drug (Dox), CXCR4-CXCL12 axis inhibitor, RT, microwave, X-Ray, chemodynamic (CDT), or sonodynamic (SDT) therapy cooperate in ICD induction mainly through HMGB, and CRT release. Their combined actions rescue DC, CD8, CD4, and memory T cells while restraining Ado support to Tregs and MDSC/M2-TAM **(A-D)**. A2AR signaling, along with coinhibitory signals such as PD-1, LAG-3, and TIM-3, limits the efficacy and fitness of CAR-Ts. A2AR gene editing, HIF-1α suppression through oxygen supply, and high INO production can enhance the efficacy of CAR-Ts. In A-E: Red arrow with bunted end shows specific inhibition resulting from drug action or disruption of a particular step **(E)**. Schematic overview illustrating the key steps (1-9) involved in the cancer vaccine workflow. This marks the itinerary of DC in antigen presentation and T cell activation leading to memory and effector responses (cytolytic killing). The regulatory loops, including CD73, A2AR, and PD-1 on various immune cells, as well as Treg-derived cytokines and chemokines, are outlined, suggesting their critical roles in tolerance and interference with vaccine efficacy at LN, blood, and tumors. These are the targets of CD73 and A2AR inhibitors and factor in tumor-directed nano-vaccines **(F)**. ICD, Immunogenic Cell Death; PDAC, Pancreatic Ductal Adenocarcinoma; GBM, Glioblastoma multiforme; BC, Carcinoma of Breast; CDT, Chemodynamic Therapy; SDT, Sonodynamic Therapy; HMGB1, High Mobility Group Box 1; HSP70, Heat Shock Protein 70; CRT, Calreticulin; Dox, Doxorubicin; ES, E-Selectin; STING, Stimulator of Interferon Genes; ARi, Adenosine Receptor inhibitors. Image created using BioRender.com.

Immunogenic cell death (ICD) induced by radiation and chemotherapy is a proven strategy that increases the chances of success of immune-based interventions (302). Anti-CD73 and anti-PD-1, in combination with RT, released ICD inducers such as HMGB1 and Hsp70. They increased CD8+ TILs, CD4, cDC, and augmented IFN-γ production while blocking Treg and cytokines such as IL-7 and IL-6. In this metastatic mice PDAC model, a single-agent anti-CD73 antibody was not adequate to elicit an optimal response (303) (**Figure 6A**). Similar coordination of T cells and NK cells (reinvigoration), TAM, Treg, and MDSC (suppression), is feasible by blocking the adenosinergic signaling after RT (304). Tumor-agonist nanoplatforms enable efficient delivery and anti-tumor effects by CD39 and CD73 inhibitors (305). Qi et al. designed a thermal-sensitive micellar formulation incorporating doxorubicin (Dox) and an A2AR antagonist (SCH-58261). This E-Selectin (ES)-modified micellar system, through leucocytes, co-delivers Dox and SCH 58261 to maximize the chemo-immunotherapeutic response. By inducing Dox-triggered immunogenic cell death (ICD), it paves the way for synergistic anti-tumor responses. Microwave radiation uncouples the drug components from the micelle. Subsequently, dying tumor cells via ICD increase the level of danger signals, such as ATP, HMGB1, and Calreticulin (CRT). CRT further provides an ‘eat me’ signal and increases the local neoantigen level for uptake by DC. A2AR antagonist coordinates in the subsequent maturation of DC, cross-presentation of tumor antigens to CTL that generate cytotoxic and memory phenotypes. A2AR antagonist in this milieu can impair Treg but protects effector and memory cells from A2AR-mediated suppression and kills tumors when rechallenged (301) (**Figure 6B**). CXCR4/CXCL12 axis promotes MDSC recruitment. A CXCR4 and A2AR targeting nanoplatform demonstrated that immune and metabolic co-regulation in the acidic tumor region of GBM can reinvigorate this TIME. X-irradiation facilitates the release of CPI-444 that blocks A2AR in CD8 and Treg, while AMD3100 perturbs CXCL12 and CXCR4 interaction in MDSC and M2-TAM. This formulation can cross the blood-brain barrier and synergizes with CRT and PD-1 blockade, which, through ICD induction, promotes DC maturation and delays T-cell exhaustion (306) (**Figure 6C**). Zhan M et al. reported a nanopotentiator drug designed by cross-linking ADA with chlorine-conjugated MnO_2_ and adding a reactive oxygen species (ROS)-cleavable linker. This nanopotentiator targets ROS and Ado high (2cm deep) primary tumors following concurrent chemo- and sonodynamic therapy. The platform releases ADA upon heightened ROS, which reduces Ado levels and protects DC, CD4, and CD8 function. It overcomes A2AR-mediated suppression of IFN-γ and TNF-α release and stimulates STING signaling and ICD by Mn^2+^ and ROS (307) (**Figure 6D**).

### 9.2 Adenosine signaling network in CAR-Ts

Besides lower response rates, the limited durability and weak memory responses of current checkpoint inhibitors call for newer cell-based immunotherapies. With FDA approval of Lifileucel (Amtagvi) as the first autologous TILs for advanced-stage melanoma, adoptive cell transfer (ACT) raises further optimism for solid lesions (299, 308–310). The key developments highlight the scope for modulating the adenosine pathway in the therapeutic engineering of CAR-Ts. A2AR gene deletion in CAR-T cells of both mice and human origins, using CRISPR/Cas9, results in improved efficacy compared to pharmacological intervention. The lack of A2AR leads to the suppression of genes induced by the A2AR signaling, restores the JAK-STAT signaling, and releases TNF-α and IFN-γ. Serial co-culture of these A2AR-deficient CAR-T cells with HER2+ cell lines enriches TIM-3+Granzyme+T cells (295). A dual knockdown of *Tim3* or *A2aR* offers response benefits in the cervical cancer xenograft model (311). There is further scope to refine CAR-Ts based on other checkpoints and mechanistic barriers (**Figure 6E**). Upstream signaling in the adenosinergic cascade presents a barrier to CAR-Ts and TILs due to its contribution to the hypoxia-Ado interface. Rationally designed CAR-T cells can mitigate these challenges (45, 46, 312) (**Figure 6E**). CAR-T cells loaded with an A2AR inhibitor through a liposomal formulation showed improved delivery of the agent to deeper TME areas. It spared CAR-Ts from A2AR-induced suppression. Phospho-CREB inhibition was highest in this CAR-T compared to free drug, CAR-T alone, or the drug and CAR-T combination (313). As discussed earlier, intracellular uptake and degradation of glutamines improve the stemness of CAR-Ts. The co-expression of CD39 and CD73 on CAR-Ts represents an exhausted state, but their deletion results in a modest anti-tumor response. In contrast, ADA metabolizes eAdo to INO. It blocks the engagement of A2AR, thereby restoring NF-κB activation, enhances the accessibility of the RUNX 1, 2 binding motif, an inducer of the memory differentiation factor, while suppressing CREB, which in turn improves the ICB effect and drives tumor killing. Glutamine, INO and lactate can regulate the T cell stemness (71, 314) (**Figure 6E**). In current clinical settings, CAR-T is often considered the last resort, primarily benefiting patients with liquid cancers. Encouragingly, the first randomized controlled trial of Claudin-18 isoform-2 specific CAR-Ts (satri-cel) showed a significant improvement of PFS in advanced gastric and gastroesophageal junction cancers (315). The perturbation of A2AR in CAR-Ts, as discussed in this section, may enhance their efficacy and yield better outcomes in solid tumors. Regardless of the modality, each CAR has specific safety concerns, including the risk of developing autoimmune diseases (AD). A conditional “off switch” or “safety switch” and control of cytokine release can avert CAR-Ts from causing damage when it becomes toxic (316, 317). It also allows the immune system to take “rest” and reinvigorate before acting on tumors. Alterations to the CAR antigen-binding domain, costimulatory, and CAR hinge and transmembrane regions can address these concerns (reviewed in 318) and can be harnessed for A2AR-driven CAR design.

### 9.3 Adenosine and metabolic immunoengineering

Metabolic immunoengineering directly targets the upstream sensing process rather than perturbing it at the pathway level (319). In this “metabolic Yin-Yang modulation,” metabolic supplementation (in CD8+TILs *in vivo*) and restriction (in ACT/CAR-T) during ex vivo preparation maximize the fitness of these cells and avert exhaustion (320). Interestingly, hypoxic exposure during the early phase of CAR-T activation equips them to kill tumors. After activation, ambient oxygen or low oxygen helps maintain the intermediate efficacy of these cells (321). Similarly, lactate and INO can increase CD8+T cells and CAR-T stemness (71, 314). In contrast, for TILs, drugs that reduce hypoxia and adenosine levels by disrupting the CD39 action and ADK, ENT1 blockade, or via increased ADA-INO levels, rescue CD8 cells from the exhaustion state (39, 73, 185).

### 9.4 Adenosine receptors and cancer vaccines

PD-1 blockade in recent years has achieved several extraordinary milestones, including a 100% response rate for MMRd-derived rectal cancers in a clinical trial (322). A prior study found that PD-1 blockade leads to the expansion of effector memory T cells in responders only (323). This expansion is consistent with the typically immunogenic profile of MMRd tumors (219, 322). The observed link between strong responses and immune richness reiterates the importance of broadening memory T cells for enduring responses (219, 324). Although CAR-T is one of the next leading modalities, it is promising primarily in liquid cancers. Moreover, scalability, safety, and cost factors limit its reach to clinically deserving patients (325, 326). In contrast, cancer vaccines can eliminate early-stage solid lesions and have shown promising results for PDAC and MSS-positive CRC (327–329). Long-lasting memory responses exhibit their curative potentials. They have a competitive advantage in terms of the breadth of the neoantigen response, which can be induced by tumor lysates, neoantigen peptides, and mRNAs (330, 331). A personalized neoantigen peptide vaccine showed durable CTL responses and epitope spreading when combined with chemotherapy and anti-PD-1 in first-line NSCLC (332). Vaccines can also boost CAR-T efficacy through exerting synergistic effects (333). Although the adenosine network garnered attention primarily for its role as an immune checkpoint and application in CAR-Ts, the insights gained from these modalities can provide valuable information beyond CAR-Ts. This insight is critical, as earlier efforts to generate DC-based cancer vaccines suffered because of tumor-intrinsic evasive factors (103). Genetic ablation of A2AR in mice can enhance the preclinical efficacy of tumor vaccines. By eliciting a robust CTL response, the irradiated GM-CSF-secreting melanoma vaccine significantly reduces the incidence of lung metastasis. It protects mice when sequentially challenged with low to high tumor loads. The mitigated adenosine signal in this situation synergizes with the B7-DC/Fc fusion protein, a DC-restricted PD-1 inhibitor (127). In the lymph nodes, antigen processing, cross-presentation, and T cell priming by neoantigen-loaded DCs face mechanistic resistance from MDSCs and Tregs. IL-10 and TGF-β, and direct A2AR and A2BR signaling derail DC and CD8+T cell interaction. In the core TME, adenosinergic and A2AR-proficient Tregs, cytokines/chemokines derived from them, along with other regulatory T cell subsets, can override CTL responses. This effect depends on the stage of the disease (31, 61, 137, 334, 335). Lymph node-guided cancer vaccines hold promise for both safety and efficacy due to their site-directed action (336). However, adenosine in LN and tumors can target diverse cell types to dampen *in situ* vaccine response. For example, a nanovaccine based on redox-sensitive nanomicelle encapsulating Dox and TLR-7/8 agonist (R848), combined with A2AR antagonist (SCH58261), mounts a two-prong attack: Dox and TLR-7 in glutathione-rich tumors destroy tumor cells and induce DC-mediated immune response. On the other hand, SCH58261, by inhibiting A2AR, blocks the suppression of NK and CD8 cells, as well as inhibits Treg expansion (337). The B-cell dysregulation by A2AR signaling impedes their homing to tumors (146). In hypoxic and Ado-rich germinal center, A2AR limits follicular helper T-cell differentiation, B cell frequency and responses, and IgG class switching, post-immunization (338, 339).

The collective knowledge gained from similar studies points out that adenosine footprints can guide the development of adjuvant cancer vaccines (**Figure 6F**). For example, inhibition of CD73 and A2AR can enhance the efficacy of DC vaccines, and CD73-directed siRNA can potentiate this effect (340, 341). Similarly, silencing A2AR in T cells enhances the efficacy of the DC vaccine, resulting in long-term protection of mice by boosting cytotoxic T cell functions (342). Adenosine also influences T helper function and TCR repertoire diversity, which could serve as critical determinants of vaccine efficacy. Sequence analysis of the TCR Vβ genes in PBMCs from patients pre- and on-ciforadenant (+anti-PD-L1) showed broadening of the TCR repertoire diversity and the treatment efficacy (204). Similarly, TCR repertoire analysis revealed that co-expression of CD73 and PD-1 on naïve CD4+T cells restricts the expansion of autoreactive T cells following exposure to self-antigens. However, foreign antigen-exposed T cells don’t express discernible PD-1 and CD73. PD-1 and CD73 co-expression on CD4+T cells differentially guides their proliferative fate when these cells are primed with self- and foreign peptides. Self-specific CD4 cells induced by the vaccine also overcome restricted expansion when CD73 and PD-1 are co-inhibited (343) (**Figure 6F**). In summary, by targeting early-stage and advanced lesions, cancer vaccines have the potential to transform the treatment landscape. A2AR and A2BR footprints in this landscape illuminate the future opportunity to design rational combinations for curative success. The key barriers to targeting adenosine signaling are summarized in **Table 3**.

**Table 3:** Key challenges related to the clinical translation of adenosine pathway inhibitors in immuno-oncology and emerging strategies to address them.

Key challenges	Mechanistic highlights	Combative strategies	References
Ligand-receptor heterogeneity	• Pan-cancer heterogeneity of *CD39, CD73*, and *ADORA2A, ADORA2B*. • Varying concentrations of eAdo in time and space. • eAdo level varies across tumor types.	• Spatial biology (single 3D cell atlas, HTAN). • Surrogates/mechanistic: pCREB, CD73, AdenoSig, NRA4. • Precision oncology functional platforms.	(8, 30, 31, 204, 219, 294, 296, 344)
Altered A2AR regulations	• A2AR and A2BR heterodimers diminish ligand affinity for A2AR. • Endosomal GPCR signaling. • ENT1 driven Ado entry to immune cells. • Spliced variants of A2AR in tumor cells worsen the disease.	• Heterodimer-targeting agents. • Degraders of CD73. • Preventing CD73 deubiquitylation. • ENT1 blockers, ADA agonists.	(39, 130, 131, 307, 345)
Constitutive A2A/A2BR signaling	• CAMs and CIM of A2AR attribute to tonic signaling. • Mutation hotspots in non-ligand binding and relatively silent regions of A2BR (gain of function). • C-terminal signaling by TREX.	• Inverse agonists. • NAMs. • A1R knock-in CAR-T • Drugs preventing tonic signaling.	(64, 82, 346–350)
ECM stiffness	• Mechanical ECM stiffness resists ECM penetration and further inward movement of TILs in highly desmoplastic tumors.	• ADC, CD39 nanobody. • ICD Induction. • CT/RT, Chemokine-OV, liposomal A2AR inhibitors. • Nanovaccine.	(140, 186, 301, 307, 337, 351)
Confounders of Ado siganling cascade	• Lactate dysregulates IFN-γ signaling in CD8 cells. • LDHA represses NFAT in NK and CD8 cells. • Kyn binding to overexpressed AhR induces ex vivo differentiation of Th0 to Treg.	• OXPHOS in CD8, oxygenation therapy, mitochondria transfer. • Metabolic immune engineering • AhR gene deletion.	(44–46, 67, 68, 194, 312, 314, 320, 352, 353)
Immune evasion and divergence	• CD39+ and CD73+ EVs. • Spread of EVs to TDLN and metastatic sites. • IL-2 is available to both CD8 and Treg for their expansion. • MTAP loss variant cancers bypass direct A2AR antagonist blocking.	• CXCR4i-A2ARi dual regulation platform. • Bifunctional TGF-β trap, Bispecific engagers. • Cis-acting IL-2, anti-CD73-IL-2v bispecific fusion proteins. • PEG-MTAP, PRMT5 inhibitors.	(55, 64, 125, 173, 249, 253, 259, 300, 354)

CAM, Constitutively Active Mutations; CIM, Constitutively Inhibiting Mutations; NAMs, Negative Allosteric Modulators; Kyn, Kynurenine; AhR, Aryl hydrocarbon Receptor; OV, Oncolytic Virus, ECM, Extracellular Matrix, EV, Extracellular Vesicles; ENT1, Equilibrated Nucleoside Transporter 1; TREX, Transcription-Export; TIME, Tumor immune microenvironment; TRIM21, Tripartite motif-containing protein 21; OTUD4, OTU Deubiquitinase 4; MTAP, Methylthioadenosine Phosphorylase; PEG-MTAP, PEGylated-MTAP; PRMT5, Protein Arginine Methyltransferase 5.

## 10 Conclusions and future direction

We described the intricacies of adenosine signaling and its multilayered immune-evasion strategy. The nexus between the adenosine axis and its metabolic regulation remodels the tumor microenvironment and T-cell exhaustion. Early clinical trials showed modest benefits as monotherapy, mainly due to stable disease or partial responses. Simplifying drug action mechanisms in a dynamic microenvironment, shorter target occupancy, and bypass mechanisms partly explain these initial setbacks (248). The insights gained from these studies rapidly shifted attention towards biomarker-guided combination, novel drug design, and delivery.

New research raised optimism for strategies that limit both Ado formation and its function in adenosine-high environments. Inhibitors of the ENT1 and MTAP loss pathway, A2AR-directed NAMs, and A1R knock-in CAR-T cells further expanded the therapeutic toolkit (39, 67, 82, 256, 259, 350). The scarcity of reagents crucial for delineating immune subset-specific A2AR and A2BR, and the shorter half-life of Ado, prompted spatial measurements of local adenosine levels and the use of exploratory biomarkers as well as signatures to refine trial designs (30, 204, 207, 295, 296). Nonetheless, spatial mapping of immune phenotypic clusters and metabolite interface, utilizing qMSI and RNAseq together, can provide multimodal, multiplex insights into biomarkers (26, 39, 40). Recent trials (NCT04969315, NCT05501054) (285, 287) are increasingly adopting such modalities, underscoring the urgent need for their validation.

The lack of congruence between the murine and human immune systems, including their checkpoint structure and function, and clonal landscape, raises questions about the derisking value of mice functional screens (282, 284, 355). Adenosine receptors are not beyond the realm of these constraints. Calls for refining preclinical models have spurred the development of sophisticated functional screening platforms, such as patient-derived ex vivo tumor fragments, tumor-immune organoids, and human tumor xenografts (HTX), reconstituted with human immune cells (217, 218, 356). Coupling these models with biomarkers and AI-guided drug design makes them amenable to boosting drug development programs. A2AR-knockout elevates the risk of autoimmunity in mice. Anti-inflammatory agents can mitigate these risks (21, 317, 357). Rotating to selective immunosuppressive therapy (SIT) before initiating immunotherapy can help patients with pre-existing autoimmune conditions (357, 358). Future research should test the efficacy of microbiota supplementation in an A2AR and Treg-deficient autoimmune scenario (16).

Our analysis raises several outstanding questions that are essential to harness forward-looking perspectives and gauge lasting clinical benefits. Multiple non-Ado metabolites, like Kyn and PGE2, also act as immunosuppressors (279, 353). What is their level of redundancy? Can other checkpoints explain the response heterogeneity of adenosine pathway inhibitors? What strategies can prevent exosomes from interfering with the prospective trial outcomes? EGFR-vIII and MSS-specific CRC enriched in AdenoSig and unique Spp1 high TAM in mCRPC, showed a likelihood of benefits from the rational combination with A2AR inhibitors. What will be the utility of these signatures for other difficult-to-treat cancer types? Nanoplatform-enabled inhibitors could synergize with A2AR pathway inhibitors and vaccines (307, 337). What benchmarks will be established to assess the design, safety, and ensure the effectiveness beyond SOCs? How can we remodel the plasticity of different Treg phenotypes, TGF-β in the TME and peripheral organs, to balance their roles in the immune response and tolerance? What will be the mechanistic underpinnings of the microbiome-immune-metabolome interface in defining A2AR/A2BR antagonist action?

Given that the A2AR antagonist expands TCR repertoire diversity, it is crucial to assess whether this blockade also promotes the homing of various T and B cell subsets and the development of tertiary lymphoid structures (204). Therapeutic cancer vaccines will benefit from these findings. Several combination approaches are currently under evaluation alongside adenosine axis inhibitors. Adopting a master protocol (or platform trial) will be suitable for conducting multi-agent studies. The FDA Modernization Act 2.0 and other strategic initiatives, together with resources such as the human tumor atlas network and precision endpoints, highlight the need to integrate these new predictive tools for the positioning of adenosine-based oncology programs (293, 344, 359, 360). Cultural mindset, stakeholders' confidence, and a collaborative ecosystem will accelerate the transition of such frameworks.

In conclusion, by synthesizing knowledge, in this review, we have expanded the space for debate and further discussion, illustrating recent advances in the adenosine pathway that can guide the successful development of immuno-oncology drug candidates. LAG-3 inhibitor and Claudin 18 isoform-specific CAR-T cells have received approval for clinical use recently (201, 315). Closer monitoring of the development of other contemporary checkpoint inhibitors will enrich this knowledge and help define future paths and the next wave of innovation.
